# The oxylipin and endocannabidome responses in acute phase *Plasmodium falciparum* malaria in children

**DOI:** 10.1186/s12936-017-2001-y

**Published:** 2017-09-08

**Authors:** Izabella Surowiec, Sandra Gouveia-Figueira, Judy Orikiiriza, Elisabeth Lindquist, Mari Bonde, Jimmy Magambo, Charles Muhinda, Sven Bergström, Johan Normark, Johan Trygg

**Affiliations:** 1 Computational Life Science Cluster (CLiC), Department of Chemistry, Umeå University, Umeå, Sweden; 2 Infectious Diseases Institute, College of Health Sciences, Makerere University, Kampala, Uganda; 3 Department of Immunology, Trinity College, Dublin, Ireland; 4 Rwanda Military Hospital, Kigali, Rwanda; 5 Department of Molecular Biology, Umeå University, Umeå, Sweden; 6 Department of Immunology and Microbiology, College of Health Sciences, Makerere University, Kampala, Uganda; 7 Laboratory for Molecular Infection Medicine Sweden (MIMS), Umeå, Sweden; 8 Umeå Center for Microbial Research, Umeå University, Umeå, Sweden; 9 Division of Infectious Diseases, Department Clinical Microbiology, Umeå University, Umeå, Sweden

**Keywords:** Oxylipins, Endocannabinoids, Malaria infection, *Plasmodium falciparum*

## Abstract

**Background:**

Oxylipins and endocannabinoids are low molecular weight bioactive lipids that are crucial for initiation and resolution of inflammation during microbial infections. Metabolic complications in malaria are recognized contributors to severe and fatal malaria, but the impact of malaria infection on the production of small lipid derived signalling molecules is unknown. Knowledge of immunoregulatory patterns of these molecules in malaria is of great value for better understanding of the disease and improvement of treatment regimes, since the action of these classes of molecules is directly connected to the inflammatory response of the organism.

**Methods:**

Detection of oxylipins and endocannabinoids from plasma samples from forty children with uncomplicated and severe malaria as well as twenty controls was done after solid phase extraction followed by chromatography mass spectrometry analysis. The stable isotope dilution method was used for compound quantification. Data analysis was done with multivariate (principal component analysis (PCA), orthogonal partial least squares discriminant analysis (OPLS-DA^®^) and univariate approaches (receiver operating characteristic (ROC) curves, t tests, correlation analysis).

**Results:**

Forty different oxylipin and thirteen endocannabinoid metabolites were detected in the studied samples, with one oxylipin (thromboxane B2, TXB_2_) in significantly lower levels and four endocannabinoids (OEA, PEA, DEA and EPEA) at significantly higher levels in infected individuals as compared to controls according to *t* test analysis with Bonferroni correction. Three oxylipins (13-HODE, 9-HODE and 13-oxo-ODE) were higher in severe compared to uncomplicated malaria cases according to the results from multivariate analysis. Observed changes in oxylipin levels can be connected to activation of cytochrome P450 (CYP) and 5-lipoxygenase (5-LOX) metabolic pathways in malaria infected individuals compared to controls, and related to increased levels of all linoleic acid oxylipins in severe patients compared to uncomplicated ones. The endocannabinoids were extremely responsive to malaria infection with majority of this class of molecules found at higher levels in infected individuals compared to controls.

**Conclusions:**

It was possible to detect oxylipin and endocannabinoid molecules that can be potential biomarkers for differentiation between malaria infected individuals and controls and between different classes of malaria. Metabolic pathways that could be targeted towards an adjunctive therapy in the treatment of malaria were also pinpointed.

**Electronic supplementary material:**

The online version of this article (doi:10.1186/s12936-017-2001-y) contains supplementary material, which is available to authorized users.

## Background

Malaria still poses a strenuous burden on the health systems in low resource countries. The most dangerous form of the disease, severe *Plasmodium falciparum* malaria, is a complex syndrome characterized by the sequestration of parasitized erythrocytes in the peripheral vasculature and a profound imbalance between pro-and anti-inflammatory responses. The acute phase response carries many common denominators with sepsis, systemic inflammatory response syndrome and in critical illness [1, 2]. However, the nature of the pathogen leaves severe malaria with certain unique features. The infection rapidly elicits complex shifts in metabolic pathways in all organ systems of the body, which leads to tissue damage [3].

Metabolic complications of malaria are increasingly recognized as contributors to severe and fatal malaria [4]. Although genomics, transcriptomics and proteomics have been used in many studies of malaria, results obtained from application of these methods do not directly reflect the changes in the metabolism of the patient.

Metabolomics, the study of small molecules, can be the most useful approach to study disease from the clinical perspective. All aforementioned methodologies, including metabolomics, are not based on any pre-assumptions and preconceptions. As such, they have the advantage of unbiased identification of disease-related signals. Initial differences in metabolite levels may be diagnostic of disease, or determinate its severity or stage [5]. As metabolite levels change over time in response to external and internal stimuli, they may also be used to follow therapeutic response and predict the clinical outcome [6, 7]. Through mapping of the perturbed metabolic levels onto the metabolic networks, it is possible to find clues to the pathogenesis of the disease.

Most of the literature reports regarding chemical analysis of plasma from humans infected with malaria deal with global screening of metabolites or lipids [8–11]. It was reported that untargeted metabolic profiling can be applied to assess the disease states in severe malaria in children [9], with fatty acids being the main class of compounds that appeared significant for grading malaria severity. In a clinical metabolomics study of sepsis [7], the metabolism of small lipid mediators stood out as the main factor differentiating the malaria sub groups. It is also known that many lipids and their derivatives produced by the parasites themselves, are immuno-active and contribute to the specific immune response seen in the malaria patient [12]. Small lipid mediators have also been found to be crucial for the initiation and resolution of inflammation during microbial infections [13, 14]. Two main classes of lipid mediators are oxylipins and endocannabinoids, where the former are oxidation products of unsaturated fatty acids, proven to be potent regulators of host immune responses [15]. Oxylipins are produced through major enzymatic pathways classified according to the enzymes involved in their production: the cytochrome p450 (CYP), cyclooxygenase (COX) and lipoxygenases 5 and 12/15 (LOX) pathways. The other major class of lipid derived mediators, endocannabinoids (eCB), activates the CB1 and CB2 cannabinoid system (CB) receptors present mainly in the brain and on immune cells respectively. The endocannabinoid receptors induce immunosuppression in a wide range of immunocytes [16]. Little is however known about levels of all these molecules in plasma during malaria infection and their relation to severity of the disease.

Small lipid mediators are found in human tissues in very low amounts (pM–nM) and hence are usually not detected with global profiling methods. An analysis of these molecules needs to be performed not only after dedicated sample clean-up, but also with specific targeted approaches. Liquid chromatography with triple quadrupe mass detector is the method most suited for analysis of very low abundant metabolites in complex matrices and hence optimal for investigation of pro- and anti-inflammatory small molecules [17].

**Fig. 1:**
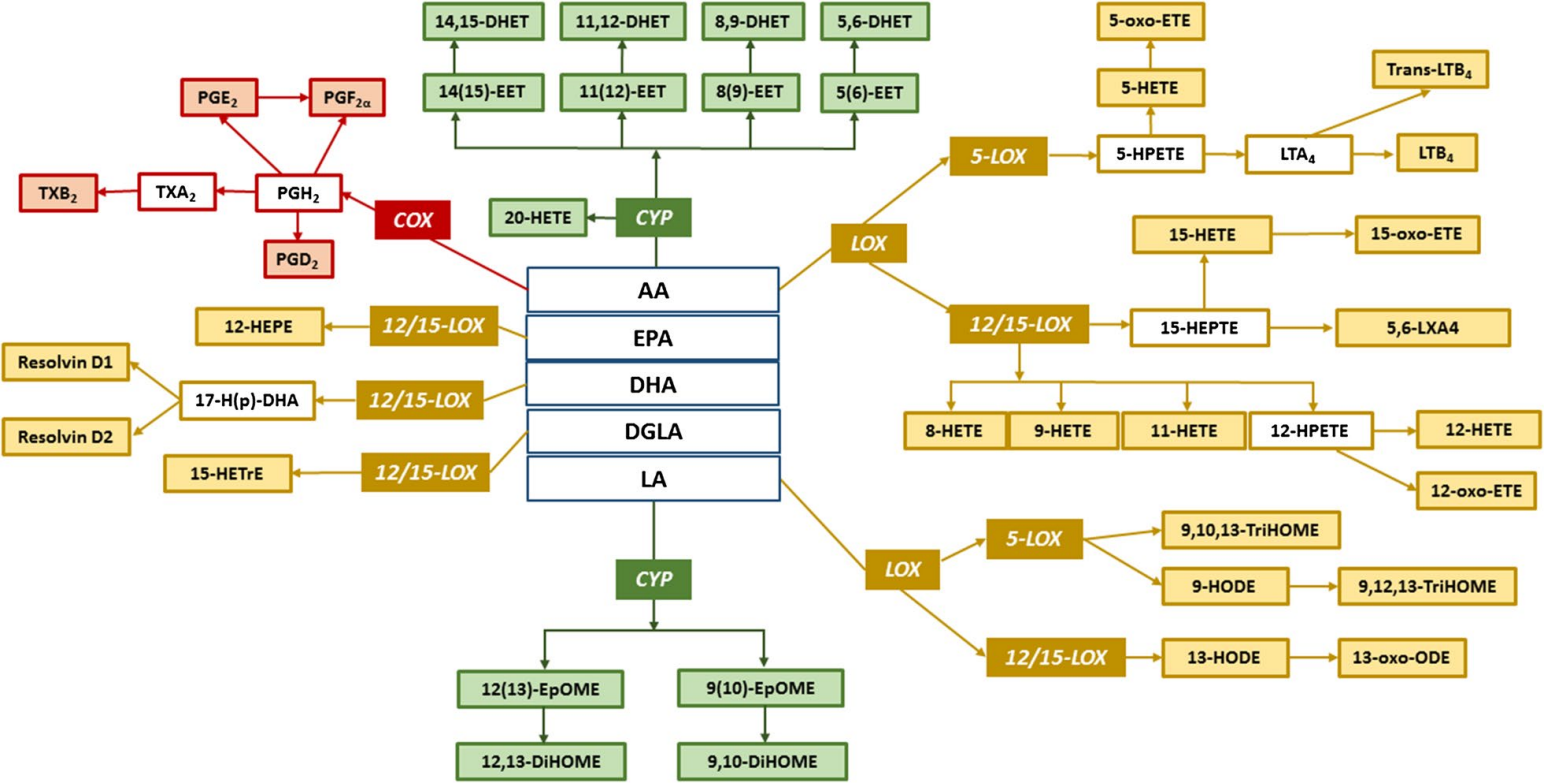
Molecules included in the study organized in biosynthetic pathways of oxylipins in humans and *coloured* according to main types of enzymes involved in their synthesis from fatty acid precursors: CYP (*green*), COX (*red*) and LOX (*yellow*); in *white compounds* that were not analysed in this project; *AA* arachidonic acid, *LA* linoleic acid, *DHA* docosahexaenoic acid, *EPA* eicosapentaenoic acid, *GLA* gamma-linolenic acid. For the simplicity of the visualization not all reactions were included in the picture. With *colour background* are marked compounds analysed within this study. Pathways are based on KEGG (http://www.genome.jp/kegg/) and BioCyc (http://www.biocyc.com/) databases

In this work a number of physiologically relevant oxylipins and endocannabinoids were quantified in plasma from malaria patients and controls with liquid chromatography tandem mass spectrometry (LC–MS/MS) approach. Investigated compounds were previously detected in plasma and belong to the main small lipid mediator synthesis pathways (Fig. 1) with known functions in inflammatory response in lungs and in diet studies [18–20]. The aim of presented study was to explore the immunoregulatory pattern of small lipid derived signalling molecules in malaria infection and to put them t into a biochemical context.

## Methods

### Patients

A total of 690 patients between 6 months and 6 years of age were enrolled from January 2011 to September 2013 at Nyagatare Hospital in Nyagatare district, Kiziguro Hospital and Ngarama Hospital in Gatsibo district or health centres in the catchment areas of the 3 hospitals in the Eastern Province of Rwanda. The patients were assessed on site by the attending paediatrician/study medical officer, and biometric and clinical parameters were recorded, as listed in Additional files 1, 2 and 3. The patients were categorized according to the WHO categories of severe malaria and uncomplicated malaria with age-matched healthy controls who were selected from healthy siblings to other kids that were seeking care in the clinical unit [21]. There were 221, 233 and 236 patients included in each group, respectively. Patients with a known HIV positive status, jaundice and with the known prior anti-malarial therapy were excluded from the study. Blood samples were drawn on site and bright field microscopy assessment of parasite presence was done in the routine lab facilities coupled to each clinic. Parasitaemia percentages were calculated by manual counting of Giemsa-stained thin blood smears.

### Patient selection

Representative individuals were chosen from the 690 samples, based on clinical information. For the lipidomics analysis, we selected 20 samples from each group of diagnostic categories (healthy controls, uncomplicated and severe malaria), 10 of each gender using a two factor full factorial design applied to principal component analysis (PCA) plots of two-component models on all available samples with clinical data as X variables [22], (Additional file 4). Because of instrumental issues (contamination of the chromatographic column which resulted in necessity to shift the analysis for 3 days to get a new column; by which time chemically unstable endocannabinoids were degraded), only 35 samples (11 controls, 13 uncomplicated and 11 severe malaria) could be analysed with the endocannabinoid method; these are also listed in Additional files 1, 2 and 3.

### Combined sample extraction for oxylipins, endocannabinoids and related lipids

Plasma was prepared on site in Rwanda and snap frozen on site in liquid nitrogen. It was stored and transported in liquid nitrogen to Sweden, thawed once for aliquoting and frozen again at −80 °C. A previously reported solid phase extraction (SPE) protocol [23] was modified to isolate oxylipins and endocannabinoids from plasma samples. In summary, extraction was performed on Waters Oasis HLB cartridges (60 mg of sorbent, 30 μm particle size). These were first washed with ethyl acetate (1 mL) and MeOH (2 × 2 mL), then conditioned with 5% MeOH in water (containing 0.1% acetic acid), before loading the sample. Quantitative volumes were used from each sample (180 μL) spiked with 10 µL internal standard (IS) mixture containing 50 ng/mL 12,13-DiHOME-d_4_ and 12(13)-EPOME-d_4_, 25 ng/mL 9-HODE-d_4_, PGE_2_-d_4_ and TXB_2_-d_4_, 800 ng/mL 2-AG-d_8_, 40 ng/mL PGF_2α_-EA-d_4_ and PGE_2_-EA-d_4_, 20 ng/mL AEA-d_8_, OEA-d_4_, SEA-d_3_ and PEA-d_4_ as well as 10 µL antioxidant solution (0.2 mg/mL BHT/EDTA in methanol/water (1:1)). After applying the sample, IS, and antioxidant solution, the SPE cartridge was washed with 5% MeOH, 0.1% acetic acid in water, dried under high vacuum and eluted with 2 mL methanol, 3 mL acetonitrile and 1 mL ethyl acetate into polypropylene tubes containing 6 µL of a glycerol solution (30% in methanol). Eluates were evaporated using a MiniVac system (Farmingdale, NY, U.S.A.), reconstituted in 100 μL of MeOH, spiked with 10 µL recovery standard (25 ng/mL CUDA), transferred to vials and analysed by LC–MS/MS.

### LC–MS/MS equipment for analysis of oxylipins, endocannabinoids and related lipids

An LC–MS/MS instrument, the Agilent Ultra-Performance (UP)LC system (Infinity 1290) coupled to an Agilent 6490 Triple Quadrupole with an electrospray ionization source (ESI) equipped with the iFunnel Technology (Agilent Technologies, Santa Clara, CA, USA), was used with separate injections for subsequent ionization in positive (endocannabinoid) and negative (oxylipin) mode. Chromatographic separation was done on a Waters BEH C_18_ column (2.1 mm × 150 mm, 130 Å, 1.7 µm particle size) with an online filter, and injection volume was 10 µL for each run. The eluents in the mobile phase consisted of (A) 0.1% acetic acid in MilliQ water and (B) acetonitrile:isopropanol (90:10). For oxylipin separation the following gradient was employed: 0.0–3.5 min 10-35% B, 3.5–5.5 min 35–40% B, 5.5–7.0 min 40–42% B, 7.0–9.0 min 42–50% B, 9.0–15.0 min 50–65% B, 15.0–17.0 min 65–75% B, 17.0–18.5 min 75–85% B, 18.5–19.5 min 85–95% B, 19.5–21 min 95–10% B, 21.0–25.0 min 10% B [24]. The separation gradient for endocannabinoids and related lipids (including prostamides) was as follows: 0.0–2.0 min 30–45% B, 2.0–2.5 min 45–79% B, 2.5–11.5 min 79% B, 11.5–12 min 79–90% B, 12–14 min 90% B, 14–14.5 min 90–79% B, 14.5–15.5 min 79% B, 15.6–19 min 30% B [17]. The last 3 min of the gradient was directed to waste to reduce MS contamination.

The electrospray ionization and mass analysis conditions were: capillary and nozzle voltage at 4000 and 1500 V, drying gas temperature 230 °C with a gas flow of 15 L/min, sheath gas temperature 400 °C with a gas flow of 11 L/min, the nebulizer gas flow was 35 psi, and iFunnel high and low pressure RF were set at 90 and 60 V (negative mode) and 150 and 60 V (positive mode). Dynamic multiple reaction monitoring (MRM) mode was used with fixed time windows (retention time ± 2 min) to profile two transitions per compound (one quantitative and one qualitative). The dynamic MRM option was performed for all compounds with optimized transitions and collision energies. The MassHunter Workstation software was used for instrument control and for manual integration of all peaks.

### Standards and calibration curve preparation

The stable isotope dilution method was used to quantify each class of bioactive lipids (oxylipins and endocannabinoids together with related lipids). Two types of internal standard were used: (i) for quantification purposes, deuterated IS was added before extraction, and (ii) for the purpose of monitoring the loss of IS, the recovery standard CUDA was added after extraction [25]. Five IS were used for quantification of endocannabinoids and related lipids (AEA-d_8_, SEA-d_3_, PEA-d_4_, OEA-d_4_ and 2-AG-d_8_), and eight for oxylipin quantification (12,13-DiHOME-d_4_, 12(13)-EpOME-d_4_, 9-HODE-d_4_, PGE_2_-d_4_, PGD_2_-d_4_, 5-HETE-d_8_, 20-HETE-d_6_ and TXB_2_-d_4_). For each native compound, a suitable IS was selected based on structural similarities (Additional file 5). Standard solutions were prepared at 10 different levels (Additional files 6, 7) to determine calibration curves by the least-squares linear regression model with equal weighting factor using the equation *y* = *mx* + *b*, where *y* corresponds to the response ratios (native standard peak area/internal standard peak area), *m* is equal to the slope of the curve, *x* corresponds to the on column concentration of the analyte and *b* is the y-interception of the calibration curve.

### Multivariate and univariate data analysis

The SIMCA software version 14.0 from MKS Umetrics AB (Umeå, Sweden) was used for all data modelling. All data was log-transformed, column mean centred and divided by its standard deviation, i.e. scaled to unit variance. principal component analysis (PCA) was used to investigate overall variability within the data and OPLS-DA [25] to model between-class differences. A summary of the studied PCA and OPLS-DA models is given in Additional files 8 and 9. In all cases seven-fold cross validation was used for model building and ANalysis of Variance testing of Cross-Validated predictive residuals (CV-ANOVA) [26] was used as diagnostic tool for assessing the reliability of the OPLS models. Metabolite significance was based on jack-knifing permutation [27]. All univariate statistics were done using the statistical package built into GraphPad Prism 6 software (San Diego, CA, U.S.A.) including: D’Agostini and Pearson omnibus normality test, receiver-operator characteristic (ROC) curves [28], t tests (parametric and non-parametric) and two-tailed Pearson correlation coefficients (r) with 95% confidence interval.

## Results

### Characterization of plasma concentration and profiles of oxylipin metabolites

Forty different oxylipin metabolites were detected in the studied samples with levels in the range of 0–98.7 nM, with 9,10-DiHOME and 12,13-DiHOME being the most abundant. The distribution of concentration levels in each of the studied groups of samples (controls, uncomplicated and severe malaria) is presented in Additional file 10 (median and interquartile range). Out of the forty compounds, 6-ketoPGF_1α_ was detected only in one control, two uncomplicated and two severe cases and 5(S)6(S)-LXA_4_ was detected in one control, two uncomplicated and three severe samples. Both compounds were hence not suitable for obtaining statistically relevant information and were removed from further data processing. Resolvin D1 displayed three values in uncomplicated malaria group, five in controls and seven in severe cases, therefore, this metabolite was also not suitable for univariate statistics, but was kept in the OPLS-DA models. Because the levels of the majority of the compounds did not follow Gaussian distribution (assessed by D’Agostini and Pearson omnibus normality test) in any of the groups studied, further analysis focused on log-transformed data. The transformed data followed normal distribution in the majority of cases and hence parametric tests could be used to study this data. The exception was 9-HODE in the control group that was further analysed by non-parametric tests.

**Fig. 2:**
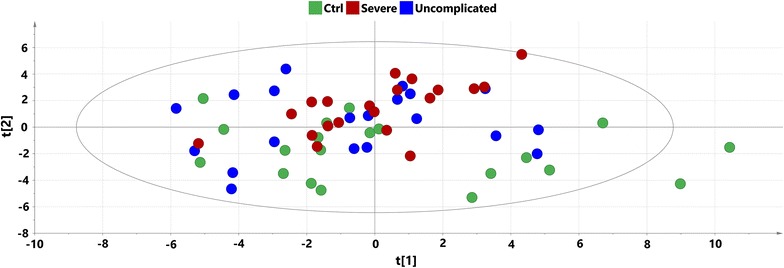
PCA score plot on oxylipin data with samples coloured according to their respective group: *red dots* signify severe malaria samples, *blue dots* uncomplicated malaria and *green* signify controls; x axis—t [1] first score (R^2^X = 0.316), y axis—t [2], second score (R^2^X = 0.170)

Principal component analysis performed on the log-transformed data was used to get an overview of the data from the 60 samples and 38 oxylipin species. On the PCA score plot (Fig. 2) partial separation between infected and non-infected individuals could be seen along the second component, with no separation between severe and uncomplicated cases. Orthogonal partial least squares discriminant analysis methodology was used to investigate differences between the studied groups of samples and to detect oxylipin profiles differentiating studied groups. It was possible to obtain valid models according to the applied cross-validation procedure for all between-group comparisons. Significant CV-ANOVA p values (p < 0.05) for separation between uncomplicated malaria and controls as well as severe malaria and controls were obtained, but insignificant (p > 0.05) for separation between severe and uncomplicated cases.

**Fig. 3:**
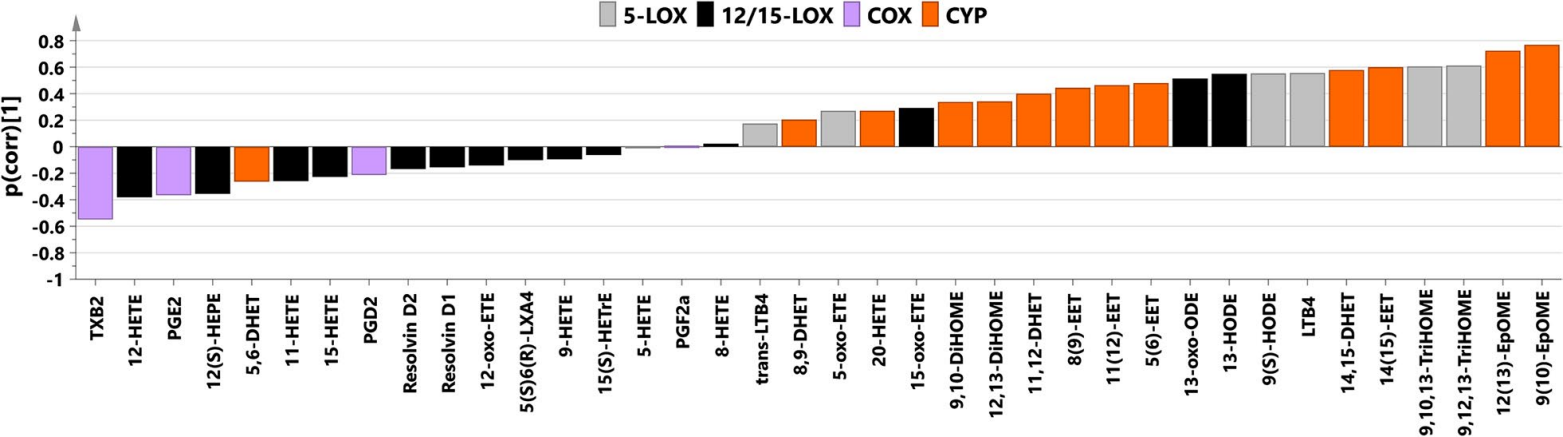
Predictive loading values (p(corr)) from the OPLS-DA oxylipin model between subjects infected with severe malaria and controls coloured according to the biochemical pathway (*grey* 5-LOX, *black* 12/15-LOX, *violet* COX, *orange* CYP); p(corr) values indicate if the metabolite is in higher or lower levels in severe malaria individuals compared with controls

**Fig. 4:**
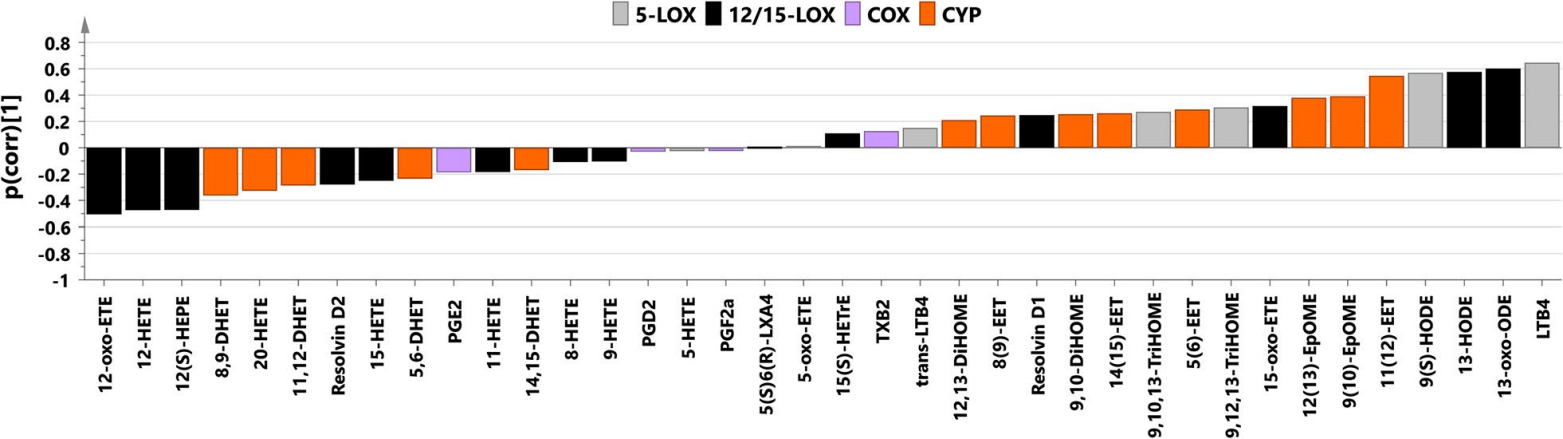
Predictive loading values (p(corr)) from the OPLS-DA oxylipin model between subjects infected with severe malaria and uncomplicated malaria coloured according to the biochemical pathway (*grey* 5-LOX, *black* 12/15-LOX, *violet* COX, *orange* CYP); p(corr) values indicate if the metabolite is in higher or lower levels in severe malaria individuals compared with uncomplicated malaria

P(corr) values from the studied models together with metabolites significant according to the jack-knifing are listed in Table 1. SUS plot [29] analysis of p(corr) vectors from uncomplicated-controls and severe-controls models revealed high correlation of their oxylipin profiles (r = 0.871, R2 = 0.76, Additional file 11), which confirms that the main discernible difference occurred between infected and non-infected individuals. Oxylipin profiles (p(corr)) for the separation between severe cases and controls and severe and uncomplicated samples are presented at Figs. 3 and 4, respectively. The main trend in the first profile was an increase of levels of compounds from the CYP and 5-LOX metabolic pathways as well as decrease of levels of metabolites from COX pathway and most of the molecules from the 12/15-LOX pathway in malaria infected individuals. The most visible trend for the separation between severe and uncomplicated cases was an increase in levels of compounds from 5-LOX pathway in severe patients compared to uncomplicated cases.

**Table 1:** Analysis of oxylipins

Compound	Abbreviation	Precursor	Pathway	OPLS-DA uncomplicated versus controls	p value uncomplicated versus controls	OPLS-DA severe versus controls	p value severe versus controls	OPLS-DA severe versus uncomplicated	p value severe versus uncomplicated
Thromboxane B2	TXB_2_	AA	COX	−*0.62*	<*0.0001*	−*0.55*	*0.0004*	0.13	NS
9,12,13-trihydroxy-octadecenoic acid	9,12,13-TriHOME	LA	5-LOX	0.36	NS	0.61	0.0022	0.31	NS
9,10,13-trihydroxy-octadecenoic acid	9,10,13-TriHOME	LA	5-LOX	0.27	NS	0.61	0.0413	0.27	NS
Prostaglandin F2aα	PGF_2α_	AA	COX	−0.10	NS	0.00	0.0056	−0.02	NS
Prostaglandin E2	PGE_2_	AA	COX	−*0.45*	NS	−0.37	0.0180	−0.19	NS
Prostaglandin D2	PGD_2_	AA	COX	−*0.38*	NS	−0.21	0.0428	−0.03	NS
Resolvin D2	Resolvin D2	DHA	12/15-LOX	0.07	NS	−0.17	NS	−0.28	NS
Resolvin D1	Resolvin D1	DHA	12/15-LOX	−0.32	–	−0.16	–	0.25	–
Lipoxin A4	5,6,-LXA_4_	AA	12/15-LOX	−0.19	NS	−0.10	NS	−0.00	NS
Trans-Leukotriene B4	Trans-LTB_4_	AA	5-LOX	−0.12	NS	0.18	NS	0.15	NS
Leukotriene B4	LTB_4_	AA	5-LOX	−0.02	NS	*0.56*	NS	*0.65*	NS
12,13-dihydroxy-octadecenoic acid	12,13-DiHOME	LA	CYP	0.25	NS	0.34	NS	0.21	NS
9,10-dihydroxy-octadecenoic acid	9,10-DiHOME	LA	CYP	0.09	NS	0.34	NS	0.26	NS
14,15-dihydroxy-eicosatrienoic acid	14,15-DHET	AA	CYP	*0.60*	NS	0.58	NS	−0.17	NS
11,12-dihydroxy-eicosatrienoic acid	11,12-DHET	AA	CYP	*0.53*	NS	0.40	NS	−0.29	NS
8,9-dihydroxy-eicosatrienoic acid	8,9-DHET	AA	CYP	0.43	NS	0.21	NS	−0.36	NS
5,6-dihydroxy-eicosatrienoic acid	5,6-DHET	AA	CYP	−0.23	0.0054	−0.26	0.0051	−*0.23*	NS
12-hydroxy-eicosapentaenoic acid	12-HEPE	EPA	12/15-LOX	−0.33	NS	−0.36	NS	−0.47	NS
20-hydroxy-eicosatetraenoic acid	20-HETE	AA	CYP	0.37	0.0017	0.27	0.0241	−0.33	NS
13-hydroxy-octadecadienoic acid	13-HODE	LA	12/15-LOX	0.06	NS	0.55	NS	0.58	0.0200
9-hydroxy-octadecadienoic acid	9-HODE	LA	5-LOX	0.09	NS	0.55	0.0350 (*)	0.57	0.0128 (*)
15-hydroxy-eicosatetraenoic acid	15-HETE	AA	12/15-LOX	−0.38	NS	−0.23	0.0402	−0.25	NS
13-oxo-octadecadienoic acid	13-oxo-ODE	LA	12/15-LOX	0.24	NS	0.51	NS	*0.60*	0.0464
11-hydroxy-eicosatetraenoic acid	11-HETE	AA	12/15-LOX	−0.37	NS	−0.26	NS	−0.19	NS
15-oxo-eicosatetraenoic acid	15-oxo-ETE	AA	12/15-LOX	−0.08	NS	0.29	NS	0.32	NS
12-hydroxy-eicosatetraenoic acid	12-HETE	AA	12/15-LOX	−0.37	NS	−0.38	0.0079	−0.47	NS
8-hydroxy-eicosatetraenoic acid	8-HETE	AA	12/15-LOX	−0.13	NS	0.02	NS	−0.11	NS
15-hydroxy-eicosatrienoic acid	15-HETrE	DGLA	12/15-LOX	−*0.27*	0.0306	−0.06	NS	0.11	NS
12-oxo-eicosatetraenoic acid	12-oxo-ETE	AA	12/15-LOX	0.01	NS	−0.14	NS	−0.51	NS
9-hydroxy-eicosatetraenoic acid	9-HETE	AA	12/15-LOX	−0.28	NS	−0.10	NS	−0.10	NS
5-hydroxy-eicosatetraenoic acid	5-HETE	AA	5-LOX	−0.14	NS	−0.01	NS	−0.03	NS
12(13)epoxy-octadecenoic acid	12(13)-EpOME	LA	CYP	*0.66*	0.0087	*0.72*	0.0017	0.38	NS
14(15)-epoxy-eicosatrienoic acid	14(15)-EET	AA	CYP	*0.60*	NS	0.60	NS	0.26	NS
9(10)epoxy-octadecenoic acid	9(10)-EpOME	LA	CYP	*0.69*	0.0107	*0.77*	0.0025	0.39	NS
11(12)-epoxy-eicosatrienoic acid	11(12)-EET	AA	CYP	0.32	NS	0.46	NS	*0.55*	NS
5-oxo-eicosatetraenoic acid	5-oxo-ETE	AA	5-LOX	0.09	NS	0.27	NS	0.01	NS
8(9)-epoxy-eicosatrienoic acid	8(9)-EET	AA	CYP	*0.44*	NS	0.44	NS	0.25	NS
5(6)-epoxy-eicosatrienoic acid	5(6)-EET	AA	CYP	0.54	NS	0.48	NS	0.29	NS

p(corr) values from the OPLS-DA models between the studied groups of samples (the changes are presented in relation to infected individuals, with minus sign showing lower and plus sign depicting higher levels in infected individuals) and summary from the univariate t test analysis with Welch’s correction with p values provided for the compounds that showed significantly different levels (p < 0.05) between studied groups of samples; in italics are marked values significant according to the OPLS-DA models (jack-knifing confidence intervals) and according to t test with Bonferroni correction for *p* = 0.05 (0.00125)

*AA* arachidonic acid, *DHA* docosahexaenoic acid, *EPA* eicosapentaenoic acid, *GLA* gamma-linolenic acid, *LA* linoleic acid

* Non-parametric Mann–Whitney test

### Univariate analysis of oxylipin levels

According to the univariate statistics (Table 1), six compounds were significant (p value below 0.05) for the separation between uncomplicated cases and controls: TXB_2_, 5,6-DHET, 20-HETE, 15-HETrE, 12(13)-EpOME and 9(10)-EpOME. The same compounds (except 15-HETrE) plus eight additional (9,12,13-TriHOME, 9,10,13-TriHOME, PGF_2α_, PGE_2_, PGD_2_, 9-HODE, 15-HETE and 12-HETE) were significantly different between severe malaria and controls. One compound, TXB_2_, passed Bonferroni correction (threshold of 0.00125) for severe-controls and uncomplicated-controls comparison. Three compounds were significantly different between severe and uncomplicated cases: 13-HODE, 9-HODE and 13-oxo-ODE, with the last showing no difference between infected and not infected individuals. A group distribution of significant compounds is presented in Additional file 12.

Correlation of oxylipin levels to the available clinical and personal descriptors of the patients’ revealed that twenty-one oxylipins were significantly positively correlated with temperature in controls, but not in infected individuals (Additional file 13).

### ROC curve analysis

Receiver-operator characteristic (ROC) curves were generated for each metabolite to test its diagnostic performance for the classification of samples into one of two groups: uncomplicated versus controls, severe versus control and severe versus uncomplicated cases. The values of the area under the curve (AUC) for all oxylipin metabolites are summarized in Additional file 14. In total nine compounds were significantly different in controls versus malaria groups (α = 0.05), none for the severe versus uncomplicated comparison, but 9-HODE showed a p value close to the set threshold (p = 0.053). From the nine metabolites significant for between group comparison based on ROC curve analysis, three were in common to uncomplicated versus control and severe versus control (TXB_2_, 5,6-DHET and 20-HETE); five were associated with severe versus control (9,12,13-TriHOME, 12-HEPE, 12-HETE, 14,15-EET and 9(10)-EpOME) and one associated with uncomplicated versus control (15-HETrE) separation.

**Fig. 5:**
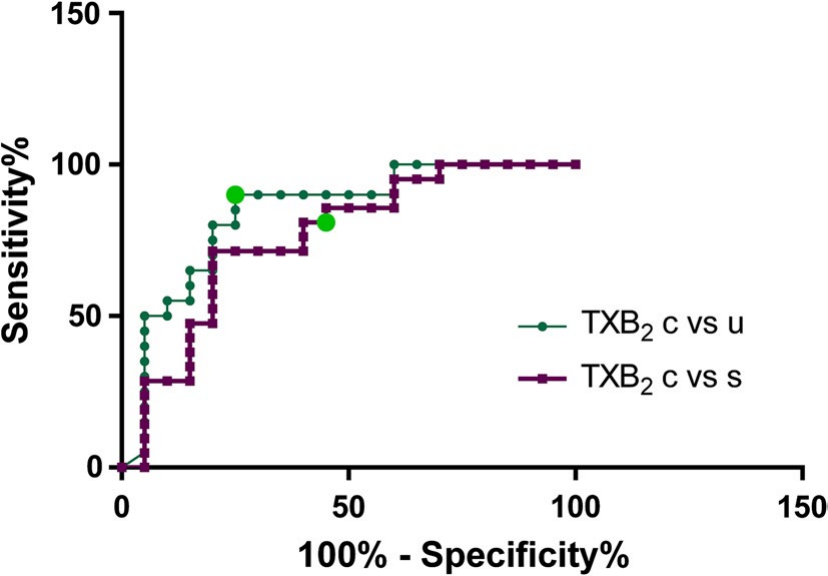
Receiver operator curves (ROC) for TXB_2_ comparing uncomplicated versus controls (*green line*) and severe versus controls (*purple line*). Youden score (<923.4 pM for uncomplicated vs controls and <609.5 pM for severe vs controls comparisons) is marked in *bright green*

Thromboxane B2 (TXB_2_) was the only compound that survived the Bonferroni cut off value (0.00125) for both severe versus controls and uncomplicated versus controls comparisons with corresponding ROC curves presented in Fig. 5. Youden score corresponds to the optimal cut off value defined as the maximum value of specificity + sensitivity-1 [30]. TXB_2_ had a Youden score < 923.4 pM (uncomplicated *vs* controls) and <609.5 pM (severe vs controls). Indeed, 18/20 of the uncomplicated cases had plasma levels lower than 923.4 pM and only 4/20 of the controls were lower than this value. For the severe cases 15/21 had levels lower than 609.5 pM whereas 4/20 of the controls had a lower value.

### Characterization of plasma concentration of endocannabinoids and related endocannabinoid profiles

A total of 15 eCB were investigated and 13 were detected at levels in the range of 0.004–253 nM with 2-arachidonyl glycerol (2-AG), palmitoyl ethanolamide (PEA) and oleoyl ethanolamine (OEA) being the most abundant. The median and range of values for all detected metabolites are given in Additional file 15. With exception of PGF_2α_-EA (present in 89% of the samples) all metabolites were detected in all samples above limit of quantification (LOQ). Given that all metabolites failed to pass D’Agostini and Pearson omnibus normality test, *log* transformation was done resulting in normally distributed data, allowing the use of parametric basal statistical tests. The exception was DHEA, which was further analysed by non-parametric tests.

**Fig. 6:**
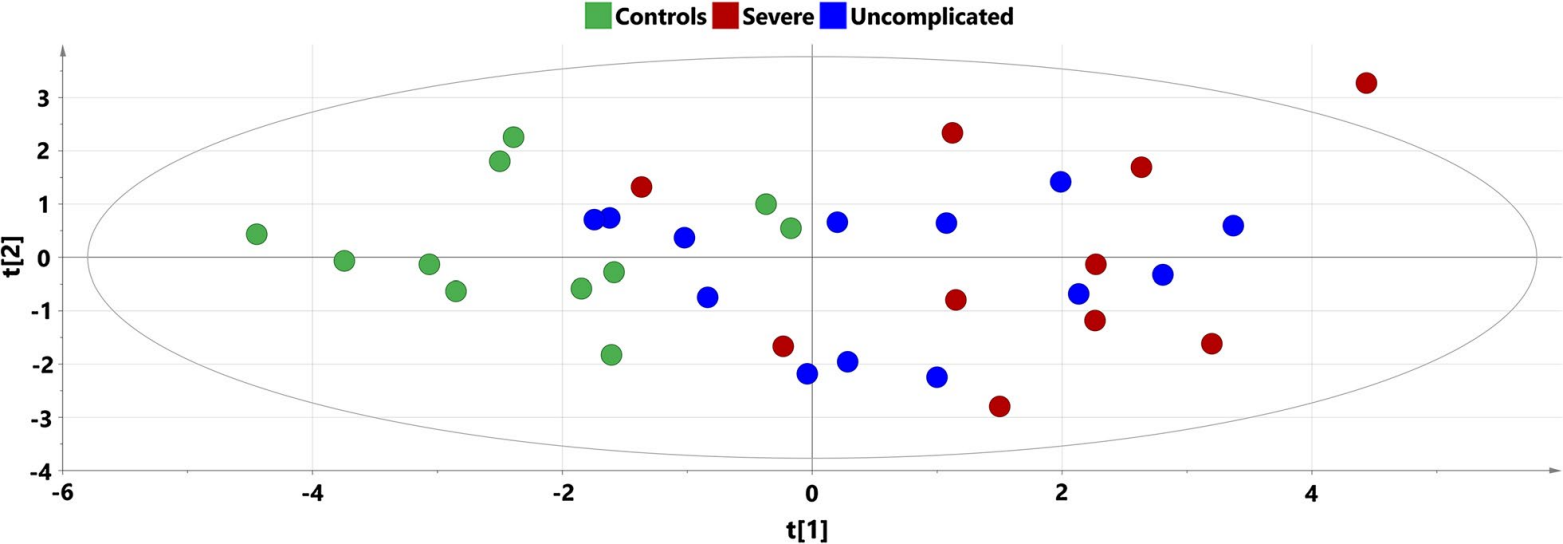
PCA score plot on endocannabinoid data with samples coloured according to their respective group: *red dots* signify severe malaria samples, *blue dots* uncomplicated malaria and *green* signify controls; x axis—t [1] first score (R^2^X = 0.413), y axis—t [2], second score (R^2^X = 0.174)

**Fig. 7:**
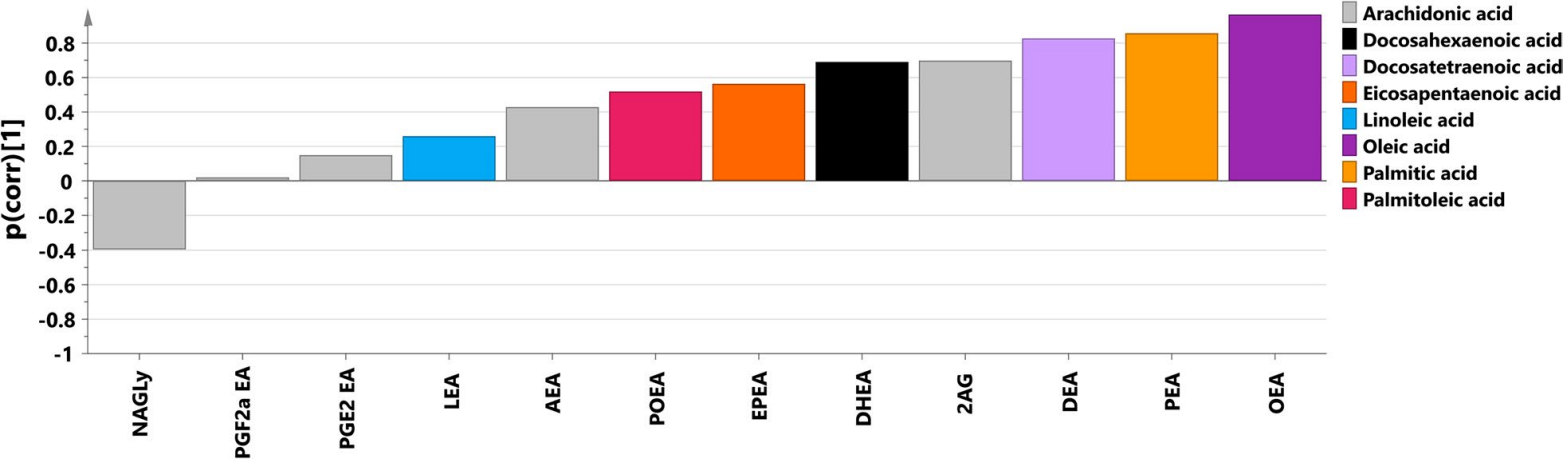
Predictive loading values (p(corr)) from the OPLS-DA endocannabinoid model between subjects infected with severe malaria and controls; endocannabinoid species coloured according to their fatty acid precursor; p(corr) values indicate if the metabolite is in higher or lower levels in severe malaria individuals compared with controls

Principal component analysis (PCA) on the log-transformed data was used to get an overview of the data from the 60 samples and 12 endocannabinoid species. On the PCA score plot (Fig. 6) clear separation between infected and non-infected individuals could be seen along the first component, with no separation between severe and uncomplicated cases. The OPLS-DA methodology was used to investigate differences between the studied groups and to detect endocannabinoid profiles characteristic for these differences. It was possible to obtain valid models according to the applied cross-validation procedure and with significant CV-ANOVA values for separation between uncomplicated malaria and controls and severe malaria and controls.

p(corr) values from the studied OPLS-DA models together with metabolites significant according to the jack-knifing are listed in Table 2. Most endocannabinoids were present at increased levels in infected individuals compared to the healthy (with exception of NAGLy for both severe and uncomplicated versus controls and PGF_2α_-EA for uncomplicated-controls comparison). SUS plot analysis of p(corr) vectors from uncomplicated-controls and severe-controls models revealed high correlation of their endocannabinoid profiles (r = 0.971, R2 = 0.94, Additional file 16). This confirms that the main difference visible was between infected and non-infected individuals. Endocannabinoid profile (p(corr)) for the separation between severe cases and controls is presented at Fig. 7.

**Table 2:** Analysis of endocannabinoids

Compound	Abbreviation	Fatty acid precursor	OPLS-DA uncomplicated versus controls	p value uncomplicated versus controls	OPLS-DA severe versus controls	p value severe versus controls
2-Arachidonoyl glycerol	2-AG	AA	*0.70*	0.0048	*0.70*	*0.0019*
Arachidonoyl ethanolamide	AEA	AA	0.20	NS	0.43	NS
Oleoyl ethanolamide	OEA	OA	*0.88*	<*0.0001*	*0.97*	<*0.0001*
Palmitoyl ethanolamide	PEA	PA	*0.80*	*0.0018*	*0.86*	*0.0001*
Docosatetraenoyl ethanolamide	DEA	DTA	*0.82*	*0.0009*	*0.83*	*0.0002*
Arachidonoyl glycine	NAGLy	AA	−0.52	NS	*−0.40*	NS
Eicosapentaenoyl ethanolamide	EPEA	EPA	*0.57*	*0.0017*(*)	*0.56*	*0.0006*
Docosahexaenoyl ethanolamide	DHEA	DHA	*0.56*	0.0046	*0.69*	*0.0025*
Palmitoleoyl ethanolamide	POEA	PO	0.57	0.0145	*0.52*	0.0224
Linolenoyl ethanolamide	LEA	LA	0.36	NS	0.26	NS
Prostaglandin F2α ethanolamide	PGF2α EA	AA	−0.28	NS	0.02	NS
Prostaglandin E2 ethanolamide	PGE2 EA	AA	0.03	NS	0.15	NS

p(corr) values from the OPLS-DA models between the studied groups of samples (the changes are presented in relation to infected individuals, with minus sign showing lower and plus sign higher levels in infected individuals) and summary from the univariate t test analysis with Welch’s correction with p values provided for the compounds that showed significantly different levels (p < 0.05) between the studied groups of samples; in italics are marked values significant according to the OPLS-DA models and according to t test with Bonferroni correction (jack-knifing confidence intervals) for *p* = 0.05 (0.0042)

*AA* arachidonic acid, *DHA* docosahexaenoic acid, *DTA* docosatetraenoic acid, *EPA* eicosapentaenoic acid, *LA* linoleic acid, *OA* oleic acid, *PA* palmitic acid, *PO* palmitoleic acid

* Non-parametric Mann–Whitney test

### Univariate analysis of endocannabinoid levels

Comparisons were made separately between controls and patients with different infection stages—uncomplicated and severe. Seven out of thirteen studied endocannabinoids were at significantly higher levels in infected individuals compared to controls; these were: 2-AG, OEA, PEA, DEA, EPEA, DHEA and POEA. From the significant metabolites, four (OEA, PEA, DEA and EPEA) passed Bonferroni correction (threshold of 0.0042) both for control versus uncomplicated and control versus severe and two metabolites (2-AG and DHEA) passed the correction only for severe-controls comparison. There were no differences in endocannabinoid levels between severe and uncomplicated malaria cases. Group distribution of significant compounds is presented in Additional file 17 in form of scatter plots.

Two compounds significantly correlated with parasitaemia scores: oleoyl ethanolamide (OEA; p = 0.015, Pearson r = 0.5485, R2 = 0.3009) and eicosapentaenoyl ethanolamide (EPEA; p = 0.040, Pearson r = 0.4747, R2 = 0.2253). The last correlation was however driven by one sample with extreme parasitaemia score and it lost significance after removal of this sample (Additional file 18).

### ROC curve analysis

Receiver-operator characteristic curves were generated for each metabolite to test its diagnostic performance for the classification of samples into one of two groups: uncomplicated versus controls, severe versus control and severe versus uncomplicated cases. The area under the ROC curves for all endocannabinoid metabolites and statistical parameters are summarized on Additional file 13. In total, seven metabolites had a p < 0.05 for differentiation between infected and non-infected individuals; none was found to be discriminant between the severe versus uncomplicated group. From mentioned seven metabolites, six (2-AG, OEA, PEA, DEA, EPEA and DHEA) were common to uncomplicated versus control and severe versus control groups. POEA significance was specific to uncomplicated versus control group.

**Fig. 8:**
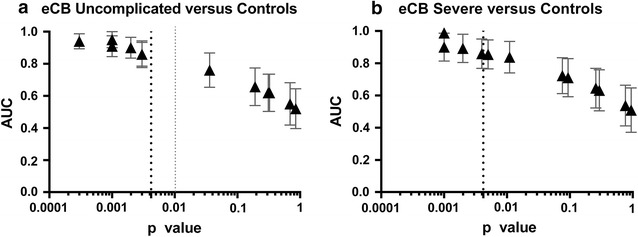
Endocannabinoids ROC curves significance. **a** The area under the ROC curve (mean ± SE) are plotted against the p values from the ROC analyses for all the endocannabinoids analysed in the groups uncomplicated malaria versus controls. The metabolites with the highest p value and area under the ROC curve (*left* to *right*) are: 2-AG, OEA, DEA, DHEA and PEA. **b** The same approach is presented for severe malaria versus control group with 2-AG, PEA and OEA with the highest p values. Both for **a** and **b**, the *dotted line* represents Bonferroni cut off (0.0042)

The significance of the ROC curves statistics for the studied endocannabinoids including the compounds that survived Bonferroni cut-off value (0.0042) for the differences between both severe versus controls (2-AG, OEA, PEA and DEA) and uncomplicated versus controls (2-AG, OEA, PEA, DEA, EPEA and DHEA) is presented at Fig. 8. Metabolites that passed Bonferroni correction had similar Youden scores both for uncomplicated versus controls and severe versus controls comparisons, which is another way of confirming that no differences in endocannabinoid levels could be seen between uncomplicated and severe malaria groups. Youden scores for all the significant metabolites are summarized on Additional file 19.

## Discussion

Oxylipin and endocannabinoid levels were analysed using two statistical approaches; univariate, in order to evaluate the statistical performance of single metabolites and multivariate in order to visualize the correlation patterns of the data and obtain compound profiles characterizing the disease that could be put into biochemical context. Whereas analysis of biomarkers is more relevant in the view of disease diagnosis and prognosis, analysis of perturbed metabolic pathways can improve our understanding of the disease itself and in the future treatment improvement.

The diagnostic properties of the studied molecules can be evaluated by p values and ROC curve analysis. In presented study, the endocannabinoid group was found to be more useful for discrimination between infected versus controls, with a total of four significant metabolites according to the t test with Bonferroni correction (OEA, PEA, DEA and EPEA). Only one significant compound was found in the oxylipin pool (TXB_2_) that was differentiating infected versus controls groups. More oxylipins were significant according to t-test without multisampling correction though, including three oxylipins (13-HODE, 9-HODE and 13-oxo-ODE) that were at significantly higher levels in severe compared to uncomplicated malaria cases. Bonferroni correction is very stringent and it has been argued if its application does not result in too low a cut-off when looking for potential biomarkers in profiling studies [31]. Whereas this issue cannot be resolved within this project, presented results point on the possibility to find within oxylipin class molecules that could differentiate between severe and uncomplicated malaria.

### Oxylipins

The acute phase response to infection initiates an interlocking system of metabolic pathways. Through observation of how representatives of the four major metabolic routes for oxylipin synthesis are affected by malaria infection, we may understand how this specific class of molecules fits into the acute phase response. Prostanoids, a subgroup of oxylipins that includes prostaglandins, prostacyclin and thromboxane, are synthesized in the body essentially by COX enzymes. There exists two classes of COX-enzymes: COX-1, which are expressed constitutively in most cells, being the dominant source of prostanoids that assist housekeeping functions, such as gastric epithelial cytoprotection and homeostasis; and COX-2, that are induced by inflammatory stimuli, hormones and growth factors and are the most important source of prostanoids in pain and inflammation [15, 32–35]. In our study, all metabolites from the COX pathway were at lower levels in malaria patients and were significantly decreased in severe patients compared to controls (Table 1), which could suggest the inhibition of action of not only COX-2, but also COX-1 enzymes in malaria infection. A lack of correlation of the levels of COX products with temperature in infected individuals, in contrary to the strong correlation observed for controls, would also support this statement. Another explanation of the reduced levels of metabolites from COX pathway in the malaria patients could be unwitnessed NSAID ingestion by patients. This is however very unlikely since the paracetamol (which is not a COX inhibitor) is the common available agent in the area used as antipyretic. Results from this study correspond to previously published data, reporting reduced PGE_2_ levels in patients with *P. falciparum* [36], but contradict the ones obtained from *Plasmodium vivax*-infected individuals, where levels were higher in infected individuals compared to uninfected, but lower in severe malaria compared to uncomplicated [37]. This relationship was also correlated to malaria severity. The proposed mechanism for lower levels of PGE_2_ in the malaria patients was haemozoin-induced suppression of COX-2 expression [38]. Suggested inhibition of the activity of COX enzymes in humans corresponds to the results from mice models, which also suggested that prostaglandins could have protective effect in murine cerebral malaria [39, 40]. This brings additional argument to the discussion raised by others regarding negative effects of COX inhibitors when used as antipyretics in acute malaria infection [41–43].

As mentioned above, TXB_2_ was the only oxylipin metabolite that passed multisampling correction test for significance (both in univariate stats and ROC curves). This means that in our study it was the most significant compound among the studied oxylipins able to differentiate between malaria patients and controls. It was present at lower levels both in severe and uncomplicated patients, a fact that contradicts previous work conducted in a *Plasmodium berghei* hamster model [44]. TXB_2_ is a non-enzymatic decomposition product of the unstable TXA_2_. It is produced by activated platelets and has prothrombotic properties. The role of platelets in immune and inflammatory cells was recently reviewed by Morell et al. [45] and blood platelet changes were observed to be of high importance during malaria infection [46, 47]. Altered platelet indices were also described to be potential marker of severe and complicated malaria caused by *P. vivax* [48]. Evidence of platelet activation was found within 24 h of infection in a mouse model, with the main effect being the reduction of parasite growth early post infection. However, as the disease progressed, continued platelet activation contributed to the development of experimental cerebral malaria (ECM) and ECM-associated inflammation [49–51]. Protective function of platelets in early stages of malaria infection could be at least partly contributed to the binding of malaria-infected red cells and killing of the parasite [52]. In this case the reduced levels of TXB_2_ may indeed reflect altered platelet function during malaria infection.

The 5-lipoxygenase (5-LOX) pathway is the major source of leukotrienes (LTs), released mainly from myeloid cells. Leukotrienes are potent pro-inflammatory and immunoregulatory lipid messengers that play a central role in immune responses and tissue homeostasis [34, 53]. They are secreted in response to a spectrum of infectious agents to enhance the capacities of macrophages and neutrophils to ingest and kill microbes and to produce antimicrobial mediators [54]. In presented study all metabolites from 5-LOX pathway were increased in severe cases compared to both controls and uncomplicated malaria patients, which could connect this pathway to the level of the inflammation in the body. Inhibition of 5-LOX and COX-2 pathways in a mouse model of sepsis reduced the inflammatory cascade associated with sepsis and improved the survival rate [55]. Absence of functional 5-LOX in mice (by its inhibition or targeted disruption of the 5-LOX gene) reduced the multiple organ injury/dysfunction caused by endotoxaemia [56]. At the same time inhibition of COX-2 pathway alone did not reduce all detrimental effects caused by uncontrolled inflammation in murine cerebral malaria [40]. As such, 5-LOX pathway can be of interest in treatment of severe malaria and complications connected to it.

The CYP pathway comprises a diverse array of membrane bound haem-iron containing enzymes that mainly are active in the liver [57]. Action of metabolites from CYP pathway can have different impact on the immune response, with the most upstream epoxyeicosatrienoic acids (EETs) or ω-HETEs, thought to be anti-inflammatory, and downstream DHETs, formed by soluble epoxide hydrolase (sEH), believed to be pro-inflammatory or inactive [15, 58]. Linoleic acid CYP products (EpOMEs) were for long time considered cytotoxic, but recent studies suggest that many of the detrimental effects were in fact due to their sEH metabolites DiHOMEs [59]. The majority of the CYP metabolites displayed higher concentrations in infected individuals. Unfortunately any further analysis of the potential implications of this finding can be performed because of a paucity of data in the literature regarding CYP metabolites and the immune system.

12/15-Lipoxygenase (12/15-LOX) plays a central role in the ‘‘class switch’’ of eicosanoid mediator biosynthesis from leukotrienes to lipoxins and resolvins, initiating the active resolution of inflammation [35]. In presented study most metabolites from 12/15-LOX pathway (except 15-oxo-ETE, 13-oxo-ODE and 13-HODE) were at lower levels in infected children compared to controls. Lipoxins have been proved to have protective effect in experimental cerebral malaria in mice [60, 61] and resolvin D2 was found to increase sepsis survival in a mouse model [61, 62]. Based on these reports, probable inhibition of the 12/15-LOX pathway could suggest a lower capability of the malaria infected individuals to resolve acute inflammation.

It is important to mention that some of the molecules included in this study can be also formed by non-enzymatic action of haemozoin, as presented by Schwarzer et al. [63], who showed that parasitized RBCs and haemozoin had large amounts of monohydroxy derivatives of poly-unsaturated fatty acids in their ester lipids. Among those metabolites 12-HETE and 15-HETE were toxic to human monocytes when supplemented at sub/low micromolar concentrations and hence could be connected to the immunodepression in malaria. It is difficult however to discuss relevance of this finding to our study, since it is not known whether the oxylipins made by haemozoin in infected erythrocytes are transported into plasma and if so, at what levels compared to their enzymatically synthesized equivalents.

### Endocannabinoids

Rather unexpectedly, we found that endocannabinoids were remarkably responsive to malarial infection with all compounds except NAGLy being in higher levels in severe malaria patients compared to controls and six out these at significance levels that passed Bonferroni correction.

A number of reports have indicated that cannabinoids suppress the antibody response of humans and animals [64, 65], with the proposed mechanism of action and potential therapeutic applications reviewed by Cabral and Griffin-Thomas [16]. There is conflicting data regarding the importance and benefits of CB receptor activation in acute phase responses. Both protective functions in relation to malaria and sepsis [66–71] and non-protective functions in relationship to sepsis [72] have been attributed to the activation of this receptor system. An explanation of these contradictory findings can be found in work by Sardinha et al. [73] who suggested that modulation of the endocannabinoid system, particularly CB_2_, dampens the inflammatory response during the early phase of sepsis. This may be beneficial in order to minimize tissue damage. Inhibition of endocannabinoid system at later stages of sepsis upregulates the inflammatory response and may help to reduce mortality by eliminating invading pathogens.

## Conclusions

This work presents evaluation of the oxylipin and endocannabinoid changes in plasma from children with malaria in comparison to healthy controls. A significant response of the endocannabinoid system to the malaria infection was observed, with two compounds from this group that could be correlated to the amount of parasite in the blood. Changes in activity of oxylipin synthesis pathways in infected individuals were also observed. Presented results point to the inhibition of COX pathway and activation of the 5-LOX in malaria infection that could be connected to dysregulation of immunological balance and subsequent negative effects for the patients. An inability of the infected patient to suppress inflammatory response was supported by the inhibition of 12/15-LOX pathway. The limitation of the study is the relatively small sample size per group (twenty individuals) and the fact that few patients had concomitant illnesses, which could have influence on the levels of investigated metabolites. Presented results provide first step towards understanding of the role of small lipid derived mediators in malaria infection and point at the possibility for using oxylipin and endocannabinoid synthesis pathways as targets in treatment of malaria.

## Additional files

**Additional file 1.** Clinical information for the control patients included in the study.

**Additional file 2.** Clinical information for the uncomplicated malaria patients included in the study.

**Additional file 3.** Clinical information for the severe malaria patients included in the study.

**Additional file 4.** Patient selection procedure.

**Additional file 5.** Native standards and corresponding internal standards used in the study.

**Additional file 6.** Standard concentrations used for calibration curves of endocannabinoids and related lipids.

**Additional file 7.** Standard concentrations (pg/mL) used for calibration curves of oxylipins.

**Additional file 8.** Parameters of models for oxylipins discussed in the study.

**Additional file 9.** Parameters of models for endocannabinoids discussed in the study.

**Additional file 10.** Oxylipin levels (pM) in the blood serum samples form controls, uncomplicated malaria and severe malaria patients.

**Additional file 11.** OPLS-DA correlation loadings from the uncomplicated versus controls and severe versus controls models.

**Additional file 12.** Scatter plots for oxylipins that showed significant p-values in between group comparisons.

**Additional file 13.** Correlation of oxylipin levels with temperature.

**Additional file 14.** ROC curves results for oxylipins.

**Additional file 15.** Endocannabinoids levels (pM) in the blood serum samples from controls, uncomplicated malaria and severe malaria patients.

**Additional file 16.** OPLS-DA correlation loadings from the uncomplicated versus controls and severe versus controls models.

**Additional file 17.** Scatter plots for endocannabinoids that showed significant p-values in between group comparisons.

**Additional file 18.** Correlation of OEA and EPEA with parasitaemia values.

**Additional file 19.** ROC curves results for endocannabinoids.

## Abbreviations

PCA: principal component analysis
OPLS-DA: orthogonal partial least squares discriminant analysis
LC–MS/MS: liquid chromatography tandem mass spectrometry detection
ROC: receiver operating characteristic
CYP: cytochrome P450
COX: cyclooxygenase
LOX: lipoxygenase
