# Supplementary material for: The oxylipin and endocannabidome responses in acute phase Plasmodium falciparum malaria in children
Source: Malar J. 2017 Sep 8;16:358. doi: 10.1186/s12936-017-2001-y (PMC5591560; doi:10.1186/s12936-017-2001-y)
Supplement: Supplementary file 1 — Additional file 1. Clinical information for the control patients included in the study. [file 12936_2017_2001_MOESM1_ESM.pdf]

Additional file 1

The oxylipin and endocannabidome responses in acute phase *Plasmodium falciparum* malaria in children

Table. Clinical information for the control patients included in the study (*). In bold marked samples analyzed for endocannabinoids content (see Methods section).

Study code	Age	Gender	Blood pressure	HC in cm	Weight in kg	Height in cm	MUAC in cm	Temperature	Pulse rate	Breathing rate	Date of recruitment
**NYC-76**	1 YR 1 MON	M	90/70	46	8	76	15	36.7	112	24	17/08/2012
**NYC-112**	1 YR 1 MON	F	100/80	51	13	89	17	37.4	98	24	22/08/2012
**NYC-117**	5 YR	F	100/85	49	20	117	16	37.2	92	24	22/08/2012
**NYC-118**	2 YR	M	110/75	51	12	98	16	37.4	102	24	22/08/2012
**NYC-123**	2 YR 6 MON	F	120/85	49	14	93	17	37	94	20	22/08/2012
**NYC-141**	3 YR	M	118/69	51	13	90	16	36.4	99	25	06/12/2012
**NYC-144**	2 YR 11 MON	F	NA	51	13	91	16	36.4	107	26	06/12/2012
**NYC-150**	2 YR 6 MON	F	NA	47	13	89	17	36.9	106	24	07/12/2012
**NYC-161**	4YR	M	100/70	50.5	13	95	15	36.3	101	12	18/04/2013
**NYC-177**	2 YR 6 M	F	100/50	47	10	87	15	36.5	120	22	24/04/2013
**NYC-178**	5 YR	M	115/90	50	15	90	17	36.36	104	34	24/04/2013
**NYC-180**	2 YR	M	90/90	48	9	84	14	36.1	100	30	25/04/2013
**NYC-184**	3 YR 7M	M	100/70	51	15	100	16	36.96	50	24	25/05/2013
**NYC-189**	4 YR	F	115/70	48.5	15	106	15.5	36.5	94	18	26/04/2013
**NYC-214**	5 YR 3 M	M	122/96	52	21	115	16	36.9	102	26	08/07/2013
**NYC-215**	5 YR 6 M	F	124/98	52	18	105	15	36.6	84	24	07/08/2013
**NYC-216**	4YR	F	108/86	51	18	105	16	36.7	94	24	07/08/2013
**NYC-231**	3 YR 6 M	F	NA	51	16	99	17	37	NA	NA	23/12/2013
**NYC-232**	4 YR	M	100/80	53	17	102	19	37	89	21	23/12/2013
**NYC-249**	6M	M	100/70	49	19	118	16	36.8	84	19	01/05/2014

(*) All children except NYC-189 were vaccinated and had negative or unknown HIV serostatus. HC - head circumference; MUAC - mid-upper arm circumference, NA- not assessed
