# Supplementary material for: The oxylipin and endocannabidome responses in acute phase Plasmodium falciparum malaria in children
Source: Malar J. 2017 Sep 8;16:358. doi: 10.1186/s12936-017-2001-y (PMC5591560; doi:10.1186/s12936-017-2001-y)
Supplement: Supplementary file 2 — Additional file 2. Clinical information for the uncomplicated malaria patients included in the study. [file 12936_2017_2001_MOESM2_ESM.pdf]

Additional file 2

The oxylipin and endocannabidome responses in acute phase *Plasmodium falciparum* malaria in children

Table. Clinical information for the uncomplicated malaria patients included in the study. In bold marked samples analyzed for endocannabinoids content (see Methods section).

Study code	Age (months)	Gender	Vaccination status	Blood pressure	HC in cm	Weight in kg	Height in cm	MUAC in cm	Temperature	Pulse rate	Breathing rate	Length of current illness in days	Parasitemia (%)	Other illness	Other symptoms	Other symptoms (2)	Treatment received before coming to hospital	Date of recruitment
**NGM-23**	33	M	NA	NA	50	10	NA	16	38.5	72	28	3	1.25	URTI+ AWD	Waterly stool	C		29/04/2011
**NYM-33**	26	M	NA	100/60	50	17	89	16	38.6	72	20	4	4.02	Flu		C	Antipyretics, cough medicine	07/05/2011
**NYM-40**	23	F	NA	98/60	49	14	87	18	38.9	72	20	2	0.58		Nausea, loss of appetite		Antipyretics	12/05/2011
**NYM-47**	13	F	NA	97/55	46	12	71	14	38.9	72	20	3	0.37	Skin lesions	Shivering, vomiting	C		19/05/2011
**NYM-55**	53	M	NA	NA	42	18	100	NA	39	100	24	2	1.39			C		09/03/2012
**NYM-56**	60	M	NA	NA	54	19	117	NA	NA	98	22	NA	0.94		Vomiting	C, S	Cotrimoxazol e	09/03/2012
**NYM-61**	45	M	NA	95/60	50.5	12	95	NA	NA	120	22	4	0.58			C		18/04/2012
**NYM-87**	60	F	NA	85/50	53.5	22	130	NA	39.54	130	36	3	1.9		Headache, anorexia	C		02/05/2012
**NYM-108**	48	F	NA	90/60	49	15	98	14	37.08	98	28	1	1.14					16/05/2012
**NYM-129**	49	M	NA	90/60	51	15	100	14	36.8	138	30	3	0.17		Abdominal pain, loss of appetite	C		27/06/2012
**NYM-142**	51	F	YES	110/80	53	17	100	15	39.1	130	26	1	3.7		Headache			04/07/2012
**NYM-147**	48	M	YES	100/50	49	11	91	13.5	36.7	120	26	1	1.38			C		06/07/2012
**NYM-151**	48	F	YES	110/80	48	15	96	17	36.3	95	20	2	NA		Headache			07/07/2012
**NYM-155**	39	M	YES	100/70	51.5	15	95	17	37.1	100	26	1	NA		Shivering, chills	C		09/07/2012
**NYM-160**	25	F	YES	85/60	48	10	80	14	37	104	24	2	0.89					11/07/2012
**NYM-183**	66	M	YES	90/55	52	18	109	15	37.5	118	20	1	2.34	Intestinal worms	Abdominal pain, headache	D, S		02/08/2012
**NYM-196**	48	M	YES	110/79	49	17	94	16	38.5	98	21	1	NA			C		05/12/2012
**NYM-211**	62	F	YES	NA	50	14	104	15	36.59	100	22	3	1.32	Tonsilitis				09/12/2012
**NYM-215**	72	F	YES	115/80	50.5	20	106	17	38.3	106	28	1	1.17			C, D, Deh, M		17/12/2012
**NYM-229**	60	F	YE S	120/7 0	5	18	113	16.5	39	170	60	2	1.3		Stomach ache		Anti-bacterial	05/03/2012

C – cough; D – diarrhea; Deh - signs of dehydration; HC - head circumference; M - dry mucus membrane; MUAC - mid-upper arm circumference; NA – not assessed; S - splenomegaly
