# Supplementary material for: The oxylipin and endocannabidome responses in acute phase Plasmodium falciparum malaria in children
Source: Malar J. 2017 Sep 8;16:358. doi: 10.1186/s12936-017-2001-y (PMC5591560; doi:10.1186/s12936-017-2001-y)
Supplement: Supplementary file 3 — Additional file 3. Clinical information for the severe malaria patients included in the study. [file 12936_2017_2001_MOESM3_ESM.pdf]

Additional file 3

The oxylipin and endocannabidome responses in acute phase *Plasmodium falciparum* malaria in children

Table. Clinical information for the severe malaria patients included in the study. In bold marked samples analyzed for endocannabinoids content (see Methods section).

Study code	Age (months)	Gender	Vaccination status	Blood pressure	HC in cm	Weight in kg	Height in cm	MUAC in cm	Temperature	Pulse rate	Breathing rate	Blantyre coma scale	Length of current illness in days	Parasitemia (%)	Other illness	Other symptoms	Other symptoms (2)	Treatment received before coming to hospital	Date of recruitment	Severe malaria subgroup (*)
**NYS-HYP-33**	51	F	NA	90/60	45	12	100	14	40.09	162	52	5	2	0.4		Abdominal pain, headache	C, Deh, N, U, (s)		16/05/2012	HYP
**NYA-SA-69**	27	M	YES	90/60	48	11	86	14	38	118	26	5	3	6.58			Deh, M, P, (s)		12/07/2012	SA
**NYS-RD-87**	36	F	YES		47	13	87	16	39.97	120	29	5	6	26.62		Prostation	D, M, P, (s)		08/12/2012	RD
**NYS-HYP-106**	56	F	YES	100/74	50.5	13	44	14.2	36.69	102	26	5	1	9.88	Typhoid fever		Deh, P, (n)		13/12/2012	HYP
**NYS-HYP-110**	18	F	YES		46	14		17.5	36.18	116	30	5	1	12.47			C, Conv, D, M, N, P, (n)		17/12/2012	HYP
**NYA-HYP-112**	48	M	YES	108/78	52	18	104	16	38.4	96	24	5	2	5.45	Traditional uvulectomy	Abdominal discomfort	P, (n)	Amoxycycline	18/12/2012	HYP
**NYS-HYP-117**	48	M	YES		50	18	103	16	39.8	110	30	5	2	15.02		Restlessnes, irritabilitys	N, P (s)		20/12/2012	HYP
**NYS-FC-121**	29	M	YES		51	13	92	13.5	35.7	140	30	5	2	17.53		Vomiting	B, Con, P, (n)		05/02/2013	FC
**NYS-HG-125**	72	F	YES	120/50	50.5	15	107	14.5	39.4	190	60	5	3	5.95		Abdominal pain	B, C, Con, Conv(1), D, (s)	Ampicilin, paracetamol	05/03/2013	HG
**NYS-PR-133**	53	M	YES	110/76	51	12	96	16	38.2	122	26	5	2	5.58		Vomiting	B, C, Conv(1), D, P, (n)	Traditional herbs	16/05/2013	PR
**NYS-RD-138**	56	M	YES	106/92	50	15	101	17	37	156	36	5	1	2.7	Pneumonia	Lethargy, abdominal pain	B, C, Deh, H, M, P, (s)		20/05/2013	RD
**NYS-FC-139**	24	M	YES	98/74	50	11	80	16	38.99	164	46	5	1	10.6	Intestinal parasites	Vomiting	B, C, Con, D, H, P, (s)		20/05/2013	FC
**NYS-CM-140**	55	M	YES	98/70	47	11	97	14	37.88	146	36	4	2	NA		Headache	B, C, Conv(3), W, (s)		20/05/2013	CM
**NYS-HYP-141**	62	F	NO	106/92	51	17	115	16		138	24	5	4	1.3	Bronchitis	Vomiting, shivers	H, S, (n)		22/05/2013	HYP
**NYS-CM-150**	55	F	YES	118/98	50	15	106	15.5	38.5	164	34	5	2	3.2	Bronchitis	Flue, abdominal pain	C, P, (n)		23/05/2013	CM
**NYS-HYP-175**	72	M	YES	118/94	50	22	113	17	39.3	160	44	5	1	1.6		Headache, abdominal pain	C		13/07/2013	HYP
**NYS-HYP-190**	66	M	YES	110/90	52	19	111	16	37.3	116	40	5	1	7.4		Headache, abdominal pain	H, J, N, P, S, U, W, (s)		08/09/2013	HYP

**NYS-RD-206**	64	F	YES	100/64	50	15	103	14	39.7	192	64	5	2	0.1		Shivers, vomiting	C, Deh, I, M, P, (s)	Traditional herbs	18/9/2013	RD
**NYS-PR-217**	64	F	NO	100/68	51	17	103	17	38.4	144	28	4	1	1.4		Headache, vomiting	H, P, S, (s)		25/9/2013	PR
**NYS-HYP-92**	46	M	YES	100/80	47	12	100	16	39.34	108	26	5	4	6.64		Prostartion	Deh, M, P, S, (s)		09/12/2012	HYP

B - breathlessness; C - cough; CM – cerebral malaria; Con - loss of conscious; Conv - convulsions (number); D - diarrhea; Deh - signs of dehydration; FC - febrile convulsions; H- hepatomegaly; HC - head circumference; HYP – hyperparasitemia; I - indrawing of intercostal spaces; J – jaundice; S - splenomegaly; M - dry mucus membrane; MUAC - mid-upper arm circumference, N - nasal flaring; NA – not assessed; P - is patient prostrated; PR – prostration; RD – respiratory distress; SA – severe anemia; U - black urine; W – wheezes; () – depth of breading (n-normal, s-shallow);
