# Supplementary material for: The oxylipin and endocannabidome responses in acute phase Plasmodium falciparum malaria in children
Source: Malar J. 2017 Sep 8;16:358. doi: 10.1186/s12936-017-2001-y (PMC5591560; doi:10.1186/s12936-017-2001-y)
Supplement: Supplementary file 4 — Additional file 4. Patient selection procedure. [file 12936_2017_2001_MOESM4_ESM.pdf]

Additional file 4

The oxylipin and endocannabidome responses in acute phase *Plasmodium falciparum* malaria in children

Patient selection procedure

We applied a multivariate design approach [1] to select samples representative for the whole cohort of patients. The multi-dimensional clinical data were used as basis for representative selection of the patients. PCA modelling was used to summarize clinical data into a low-dimensional hyper plane, which was visualized as a score scatter plot. Two factorial, full factorial design was applied to Principal Component Analysis (PCA) plots of two-component models with clinical data as X variables. In each of the four corners of the scatter plot, two samples (patients) were selected along with two centre points. This procedure was repeated for each gender. In this way we selected representative samples of the multivariate space (defined as samples and clinical variables related to them). Variables that were not numerical were transformed into discrete. Only variables that differed between samples and contained more than two values different from median were used. Patients presenting with jaundice and those with a known history of antimalarial treatment before sampling were excluded from the selection process. Age, height, weight and head circumference were highly correlated to each other and caused bias in the models, hence only age was included in the full factorial design selection of samples. This was also the case regarding systolic and diastolic blood pressure where only systolic pressure was included.

References:

1. Eriksson L.; Johansson E.; Kettaneh-Wold, N.; Wikstrom, C.; Wold, S. Design of experiments. Principles and applications. Third ed. Umea: UMETRICS AB, **2008**.
