# Supplementary material for: The oxylipin and endocannabidome responses in acute phase Plasmodium falciparum malaria in children
Source: Malar J. 2017 Sep 8;16:358. doi: 10.1186/s12936-017-2001-y (PMC5591560; doi:10.1186/s12936-017-2001-y)
Supplement: Supplementary file 5 — Additional file 5. Native standards and corresponding internal standards used in the study. [file 12936_2017_2001_MOESM5_ESM.pdf]

Additional file 5

The oxylipin and endocannabidome responses in acute phase *Plasmodium falciparum* malaria in children

Table. Native standards and corresponding internal standards used in the study.

Internal standard	Native standard
Endocannabinoid sand related lipids	
2-AG-d_8	2-AG; 2-LG
AEA-d_4	AEA
OEA-d_4	OEA, EPEA, NAGly, POEA, DHEA
PEA-d_4	PEA, DEA, LEA
SEA-d_3	SEA
$PGF_{2\alpha}$-EA-d_4	$PGF_{2\alpha}$-EA
PGE_2-EA-d_4	PGE_2-EA
Oxylipins	
TXB_2-d_4	TXB_2, 9,12,13-TriHOME, 9,10,13-TriHOME,
12,13-DIHOME-d_4	12,13-DiHOME, 9,10-DiHOME, 14,15-DHET, 11,12-DHET, 8,9-DHET, 5,6-DHET
12(13)-EPOME-d_4	12(13)-EPOME, 9(10)-EPOME
9-HODE-d_4	13-HODE, 9-HODE, 17-HDoHE, 13-oxo-ODE, 15-oxo-ETE, 9-oxo-ODE, 15-HETrE, 12-oxo-ETE, 5-oxo-ETE
5-HETE-d_8	5-HETE, 12-HETE, 8-HETE, 9-HETE, 14(15)-EET, 11(12)-EET, 8(9)-EET, 5(6)-EET
20-HETE-d_6	12-HEPE, 20-HETE, 15-HETE, 11-HETE
PGE_2-d_4	$PGF_{2\alpha}$, PGE_2, Resolvin D_1, Resolvin D_2, LTB_4
PGD_2-d_4	PGD_2
