# Supplementary material for: The oxylipin and endocannabidome responses in acute phase Plasmodium falciparum malaria in children
Source: Malar J. 2017 Sep 8;16:358. doi: 10.1186/s12936-017-2001-y (PMC5591560; doi:10.1186/s12936-017-2001-y)
Supplement: Supplementary file 6 — Additional file 6. Standard concentrations used for calibration curves of endocannabinoids and related lipids. [file 12936_2017_2001_MOESM6_ESM.pdf]

Additional file 6

The oxylipin and endocannabidome responses in acute phase *Plasmodium falciparum* malaria in children

Table. Standard concentrations (ng/mL) used for calibration curves of endocannabinoids and related lipids. Stock solutions for all standards were weekly prepared and stored in methanol at -80 °C. Each native standard stock solution was diluted with methanol at ten different calibration levels (S1 – S10) and stored at -80 °C.

	**Standard concentration (ng/mL)**				
	2-AG, 2-LG	POEA, LEA, AEA, DHEA, NAGly, DEA, EPEA	OEA, PEA	SEA	$PGF_{2\alpha}$-EA, PGE_2-EA
**S1**	83.3	4.17	2.08	6.94	0.67
**S2**	41.6	2.08	1.04	3.47	0.33
**S3**	20.8	1.04	0.52	1.74	0.17
**S4**	10.4	0.52	0.26	0.87	0.08
**S5**	5.21	0.26	0.13	0.43	0.04
**S6**	2.60	0.13	0.07	0.22	0.02
**S7**	1.30	0.07	0.03	0.11	0.01
**S8**	0.26	0.01	0.01	0.02	0.01
**S9**	0.05	0.003	0.0013	0.004	0.001
**S10**	0.01	0.0003	0.0001	0.0004	0.0002
