# Supplementary material for: The oxylipin and endocannabidome responses in acute phase Plasmodium falciparum malaria in children
Source: Malar J. 2017 Sep 8;16:358. doi: 10.1186/s12936-017-2001-y (PMC5591560; doi:10.1186/s12936-017-2001-y)
Supplement: Supplementary file 7 — Additional file 7. Standard concentrations (pg/mL) used for calibration curves of oxylipins. [file 12936_2017_2001_MOESM7_ESM.pdf]

Additional file 7

The oxylipin and endocannabidome responses in acute phase *Plasmodium falciparum* malaria in children

Table. Standard concentrations (pg/mL) used for calibration curves of oxylipins. Stock solutions for all standards were monthly prepared and stored in methanol at -80 °C. Each native standard stock solution was diluted with methanol at ten different calibration levels (S1 – S10) and stored at -80 °C.

	**Standard concentration (pg/mL)**
**S1**	16450
**S2**	8220
**S3**	4110
**S4**	2060
**S5**	1030
**S6**	514
**S7**	257
**S8**	129
**S9**	64
**S10**	37
