# Supplementary material for: The oxylipin and endocannabidome responses in acute phase Plasmodium falciparum malaria in children
Source: Malar J. 2017 Sep 8;16:358. doi: 10.1186/s12936-017-2001-y (PMC5591560; doi:10.1186/s12936-017-2001-y)
Supplement: Supplementary file 8 — Additional file 8. Parameters of models for oxylipins discussed in the study. [file 12936_2017_2001_MOESM8_ESM.pdf]

Additional file 8

The oxylipin and endocannabidome responses in acute phase *Plasmodium falciparum* malaria in children

Table. Parameters of models for oxylipins discussed in the study.

Parameter	PCA	OPLS-DA uncomplicated versus controls	OPLS-DA severe versus controls	OPLS-DA severe versus uncomplicated
Number of components	2	1+1+0	1+1+0	1+1+0
P1 (predictive variation)	-	12.7%	16.1%	9.9%
R2X(cum)	0.49	0.47	0.44	0.41
R2(cum)	-	0.59	0.59	0.41
Q2(cum)	0.38	0.35	0.31	0.09
CV-ANOVA	-	4.1×10^{-3}	9.7×10^{-3}	0.45
