# Supplementary material for: The oxylipin and endocannabidome responses in acute phase Plasmodium falciparum malaria in children
Source: Malar J. 2017 Sep 8;16:358. doi: 10.1186/s12936-017-2001-y (PMC5591560; doi:10.1186/s12936-017-2001-y)
Supplement: Supplementary file 9 — Additional file 9. Parameters of models for endocannabinoids discussed in the study. [file 12936_2017_2001_MOESM9_ESM.pdf]

Additional file 9

The oxylipin and endocannabidome responses in acute phase *Plasmodium falciparum* malaria in children

Table. Parameters of models for endocannabinoids discussed in the study.

Parameter	PCA	OPLS-DA uncomplicated versus controls	OPLS-DA severe versus controls	OPLS-DA severe versus uncomplicated
Number of components	2	1+1+0	1+1+0	No model
P1 (predictive variation)	-	33.8%	36.1%	
R2X(cum)	0.59	0.57	0.53	
R2(cum)	-	0.69	0.82	
Q2(cum)	0.23	0.53	0.59	
CV-ANOVA	-	4.3×10^{-3}	4.6×10^{-3}	
