# Supplementary material for: The oxylipin and endocannabidome responses in acute phase Plasmodium falciparum malaria in children
Source: Malar J. 2017 Sep 8;16:358. doi: 10.1186/s12936-017-2001-y (PMC5591560; doi:10.1186/s12936-017-2001-y)
Supplement: Supplementary file 10 — Additional file 10. Oxylipin levels (pM) in the blood serum samples form controls, uncomplicated malaria and severe malaria patients. [file 12936_2017_2001_MOESM10_ESM.pdf]

Additional file 10

The oxylipin and endocannabidome responses in acute phase *Plasmodium falciparum* malaria in children

Table. Oxylipin levels (pM) in the blood serum samples form controls, uncomplicated malaria and severe malaria patients. Shown are medians interquartile range (IQR) and total ranges for the lipids.

	Controls (n=20)			Uncomplicated malaria (n=20)			Severe malaria (n=21)		
**Compound**	**Median**	**IQR**	**Range**	**Median**	**IQR**	**Range**	**Median**	**IQR**	**Range**
**6-keto$PGF_{1\alpha}$**	0	0	0-5791	0	0	0-329	0	0	0-215
**TXB_2**	1838	17492	0-81519	73	522	0-2715	345	1184	6-10366
**9,12,13-TriHOME**	2173	1707	712-22106	3142	8136	942-22141	7627	8962	1462-22704
**9,10,13-TriHOME**	711	651	295-7596	879	777	380-6666	1260	2040	489-5450
**$PGF_{2\alpha}$**	264	1114	0-3754	291	219	0-435	159	158	0-418
**PGE_2**	188	1163	12-13266	117	540	0-2728	118	206	7-723
**PGD2**	113	1366	0-18123	99	538	0-2078	85	106	0-602
**Resolvin D2**	151	288	0-7461	327	437	0-1331	192	171	0-1942
**Resolvin D1**	0	6	0-477	0	0	0-15	0	7	0-12
**5(S)6(R)-LXA4**	74	678	0-7219	53	326	0-690	49	78	0-1044
**5(S)6(S)-LXA4**	0	0	0-109	0	0	0-594	0	0	0-1285
***trans*-LTB4**	26	289	0-18988	10	126	0-358	50	109	0-182
**LTB4**	0	8	0-64	0	0	0-42	24	49	0-286
**12,13-DiHOME**	58497	68737	15696-551254	69418	151905	12986-665258	94261	49076	16246-458524
**9,10-DiHOME**	83027	136497	15666-512201	79811	146439	11226-271801	102191	180587	12055-334198
**14,15-DHET**	9538	10696	4281-34604	13652	13312	528-124994	13544	10597	6909-37139
**11,12-DHET**	6778	9919	2895-27982	9165	11282	3373-74461	8681	9844	3142-24196
**8,9-DHET**	7218	11553	812-21858	8337	12639	2003-50746	6992	6629	2662-19355
**5,6-DHET**	7067	7881	1392-34250	2531	4031	1090-15495	3841	2849	0-10180
**12-HEPE**	1345	4282	55-45642	741	3669	105-11338	602	753	43-2704
**20-HETE**	1261	1336	0-2794	2664	2322	907-4623	2268	2459	230-6780
**13-HODE**	32442	48737	6641-554754	46196	51382	6126-105804	59477	107680	11188-334527
**9-HODE**	19804	28629	3143-635378	25931	33296	3688-71593	39696	53556	6273-185003
**15-HETE**	1944	7820	372-40927	1485	1569	289-12567	1496	1381	599-4118
**13-oxo-ODE**	7582	7756	0-45567	7714	7952	996-24260	13722	22511	1174-152879
**11-HETE**	550	3643	110-14436	337	897	40-4009	527	382	125-1574
**15-oxo-ETE**	231	829	67-6007	264	441	99-4559	358	814	111-4033
**12-HETE**	3577	20285	204-346882	298	8120	298-16044	1582	1787	359-6637
**8-HETE**	355	1377	57-6670	377	702	84-1651	430	315	165-1050
**15-HETrE**	333	2266	0-21027	250	387	0-2091	359	309	0-1377
**12-oxo-ETE**	611	6757	0-14017	1351	8060	0-16421	0	7980	0-13770
**9-HETE**	1209	13415	346-89276	1639	3565	271-14450	2052	2626	226-9485
**5-HETE**	1562	4189	173-52632	935	1698	171-4290	1325	1297	221-4193
**12(13)-EpOME**	503	810	123-9425	1584	1524	278-11671	1769	3949	418-13525
**14(15)-EET**	71	270	0-2428	172	515	0-1803	424	631	0-1249
**9(10)-EpOME**	3870	9045	969-80309	12510	20731	1615-117389	15614	29136	2392-234289
**11(12)-EET**	137	526	0-1508	187	327	0-1299	231	339	54-927

**5-oxo-ETE**	273	634	0-2512	231	388	0-1371	396	693	0-5132
**8(9)-EET**	109	286	0-974	180	326	0-1056	250	376	0-1247
**5(6)-EET**	212	300	0-3225	393	802	50-3431	690	916	106-2213
