# Supplementary material for: The oxylipin and endocannabidome responses in acute phase Plasmodium falciparum malaria in children
Source: Malar J. 2017 Sep 8;16:358. doi: 10.1186/s12936-017-2001-y (PMC5591560; doi:10.1186/s12936-017-2001-y)
Supplement: Supplementary file 13 — Additional file 13. Correlation of oxylipin levels with temperature. [file 12936_2017_2001_MOESM13_ESM.pdf]

Additional file 13

The oxylipin and endocannabidome responses in acute phase *Plasmodium falciparum* malaria in children

Table. Correlation of oxylipin levels with temperature; r – Pearson correlation coefficient, p – two-tailed p value; ns – p value above 0.05.

Compound	Controls		Infected individuals	
TXB_2	0.257	ns	-0.203	ns
9,12,13-TriHOME	0.665	0.001	0.122	ns
9,10,13-TriHOME	0.628	0.003	0.254	ns
$PGF_{2\alpha}$	0.634	0.003	-0.133	ns
PGE_2	0.555	0.011	-0.290	ns
PGD_2	0.606	0.005	-0.291	ns
Resolvin D2	0.553	0.012	-0.344	0.037
Resolvin D1	0.425	ns	-0.307	ns
5(S)6(R)-LXA_4	0.654	0.002	-0.179	ns
trans-LTB_4	0.597	0.006	-0.213	ns
LTB_4	-0.103	ns	0.225	ns
12,13-DiHOME	0.604	0.005	-0.154	ns
9,10-DiHOME	0.334	ns	-0.162	ns
14,15-DHET	0.009	ns	-0.192	ns
11,12-DHET	0.001	ns	-0.222	ns
8,9-DHET	0.368	ns	-0.254	ns
5,6-DHET	0.561	0.010	-0.268	ns
12-HEPE	0.025	ns	-0.203	ns
20-HETE	-0.078	ns	0.114	ns
13-HODE	0.572	0.008	0.109	ns
9-HODE	0.504	0.023	0.079	ns
15-HETE	0.616	0.004	-0.205	ns
13-oxo-ODE	0.454	0.044	0.033	ns
11-HETE	0.560	0.010	-0.233	ns
15-oxo-ETE	0.526	0.017	-0.183	ns
12-HETE	0.229	ns	-0.185	ns
8-HETE	0.677	0.001	-0.177	ns
15-HETrE	0.521	0.019	-0.163	ns
12-oxo-ETE	0.366	ns	0.096	ns
9-HETE	0.654	0.002	-0.234	ns
5-HETE	0.640	0.002	-0.113	ns
12(13)-EpOME	-0.252	ns	0.295	ns
14(15)-EET	-0.199	ns	0.275	ns
9(10)-EpOME	-0.229	ns	0.267	ns
11(12)-EET	-0.127	ns	0.195	ns
5-oxo-ETE	0.588	0.006	0.162	ns
8(9)-EET	-0.193	ns	0.175	ns
5(6)-EET	-0.251	ns	0.108	ns
