# Supplementary material for: The oxylipin and endocannabidome responses in acute phase Plasmodium falciparum malaria in children
Source: Malar J. 2017 Sep 8;16:358. doi: 10.1186/s12936-017-2001-y (PMC5591560; doi:10.1186/s12936-017-2001-y)
Supplement: Supplementary file 14 — Additional file 14. ROC curves results for oxylipins. [file 12936_2017_2001_MOESM14_ESM.pdf]

Additional file 14

The oxylipin and endocannabidome responses in acute phase *Plasmodium falciparum* malaria in children

Table. ROC curves results for oxylipins: area under the curve (AUC), standard error (std. error), p value and Youden score (pM), in bold significant metabolites.

	Controls *vs* Uncomplicated					Controls *vs* severe					Uncomplicated *vs* Severe			
Compound	AUC	Std error	95% CI	P value	Youden Score (pM)	AUC	Std error	95% CI	P value	Youden Score	AUC	Std error	95% CI	P value
TXB_2	0.8413	0.06560	0.7127 to 0.9698	**0.0002246**	< 923.4	0.7595	0.07706	0.6084 to 0.9106	**0.004488**	< 609.5	0.6095	0.09034	0.4324 to 0.7866	0.2303
9,12,13-TriHOME	0.6300	0.08946	0.4546 to 0.8054	0.1596		0.7452	0.08348	0.5816 to 0.9089	**0.007245**		0.6167	0.09168	0.4369 to 0.7964	0.2013
9,10,13-TriHOME	0.5381	0.09230	0.3571 to 0.7190	0.6765		0.6916	0.08528	0.5244 to 0.8588	0.03358		0.6595	0.08853	0.4860 to 0.8331	0.08061
$PGF_{2\alpha}$	0.5525	0.09698	0.3624 to 0.7426	0.5700		0.6107	0.09214	0.4301 to 0.7913	0.2253		0.6012	0.09259	0.4197 to 0.7827	0.2677
PGE_2	0.6450	0.08900	0.4705 to 0.8195	0.1167		0.6512	0.08763	0.4794 to 0.8230	0.09774		0.5143	0.09492	0.3282 to 0.7004	0.8757
PGD_2	0.6125	0.09193	0.4323 to 0.7927	0.2236		0.5750	0.5750	0.3940 to 0.7560	0.4114		0.5262	0.09741	0.3352 to 0.7172	0.7742
Resolvin D2	0.6450	0.09210	0.4644 to 0.8256	0.1167		0.5500	0.09344	0.3668 to 0.7332	0.5839		0.619	0.09081	0.4410 to 0.7971	0.1923
Resolvin D1														

5(S)6(R)-LXA4	0.5813	0.5813	0.3994 to 0.7631	0.3794		0.5917	0.08975	0.4157 to 0.7676	0.3154		0.5083	0.09382	0.3244 to 0.6923	0.9273
trans-LTB4	0.5475	0.09279	0.3656 to 0.7294	0.6073		0.5548	0.09473	0.3691 to 0.7405	0.5486		0.5905	0.09443	0.4053 to 0.7756	0.3217
12,13-DiHOME	0.5425	0.09297	0.3602 to 0.7248	0.6456		0.6095	0.09141	0.4303 to 0.7887	0.2303		0.5405	0.09346	0.3572 to 0.7237	0.6575
9,10-DiHOME	0.5625	0.09231	0.3815 to 0.7435	0.4989		0.531	0.09182	0.3509 to 0.7110	0.7346		0.5881	0.08991	0.4118 to 0.7644	0.3346
14,15-DHET	0.6675	0.08644	0.4980 to 0.8370	0.06999		0.6571	0.08756	0.4855 to 0.8288	0.08524		0.5167	0.09236	0.3356 to 0.6977	0.8551
11,12-DHET	0.625	0.08988	0.4488 to 0.8012	0.1763		0.5476	0.09282	0.3657 to 0.7296	0.6019		0.5952	0.08919	0.4204 to 0.7701	0.2969
8,9-DHET	0.5625	0.09212	0.3819 to 0.7431	0.4989		0.5	0.09382	0.3161 to 0.6839	1		0.5643	0.09225	0.3834 to 0.7451	0.4813
5,6-DHET	0.7475	0.07991	0.5908 to 0.9042	**0.007431**	< 5891	0.7786	0.07518	0.6312 to 0.9260	**0.002288**	< 5028	0.5143	0.09411	0.3298 to 0.6988	0.8757
12-HEPE	0.59	0.09112	0.4114 to 0.7686	0.3302		0.6881	0.08609	0.5193 to 0.8569	**0.0394**	< 1475	0.5762	0.09415	0.3916 to 0.7608	0.404
20-HETE	0.7975	0.06909	0.6621 to 0.9329	**0.001295**	> 2803	0.706	0.08255	0.5441 to 0.8678	**0.02411**	> 2797	0.5381	0.09181	0.3581 to 0.7181	0.6765
13-HODE	0.515	0.09505	0.3287 to 0.7013	0.8711		0.6286	0.08914	0.4538 to 0.8033	0.1591		0.6619	0.08601	0.4933 to 0.8305	0.07619
9-HODE	0.525	0.0932	0.3423 to 0.7077	0.7868		0.6643	0.08738	0.4930 to 0.8356	0.07197		0.6762	0.08458	0.5104 to 0.8420	0.05365

15-HETE	0.6425	0.08832	0.4693 to 0.8157	0.1232		0.65	0.08824	0.4770 to 0.8230	0.1004		0.5119	0.09266	0.3302 to 0.6936	0.8962
13-oxo-ODE	0.5025	0.09308	0.3200 to 0.6850	0.9784		0.6333	0.08989	0.4571 to 0.8096	0.1442		0.631	0.09019	0.4541 to 0.8078	0.1515
11-HETE	0.6425	0.08872	0.4686 to 0.8164	0.1232		0.5905	0.09381	0.4066 to 0.7744	0.3217		0.55	0.09521	0.3634 to 0.7366	0.5839
15-oxo-ETE	0.525	0.0932	0.3423 to 0.7077	0.7868		0.5405	0.09241	0.3593 to 0.7216	0.6575		0.5714	0.09057	0.3939 to 0.7490	0.434
12-HETE	0.615	0.09092	0.4368 to 0.7932	0.2134		0.731	0.07946	0.5752 to 0.8867	**0.01144**	< 3737	0.6548	0.08788	0.4825 to 0.8270	0.09007
8-HETE	0.5125	0.09471	0.3268 to 0.6982	0.8924		0.5071	0.09877	0.3135 to 0.7008	0.9376		0.5119	0.09503	0.3256 to 0.6982	0.8962
15-HETrE	0.6925	0.08465	0.5266 to 0.8584	**0.03731**	< 1523	0.5667	0.09363	0.3831 to 0.7502	0.4652		0.6095	0.09093	0.4313 to 0.7878	0.2303
12-oxo-ETE	0.595	0.09108	0.4164 to 0.7736	0.304		0.5262	0.0916	0.3466 to 0.7058	0.7742		0.6214	0.08986	0.4453 to 0.7976	0.1835
9-HETE	0.595	0.09314	0.4124 to 0.7776	0.304		0.5548	0.09534	0.3678 to 0.7417	0.5486		0.5095	0.09306	0.3271 to 0.6920	0.9169
5-HETE	0.6475	0.08833	0.4743 to 0.8207	0.1106		0.631	0.08799	0.4584 to 0.8035	0.1515		0.5262	0.09343	0.3430 to 0.7093	0.7742
12(13)-EpOME	0.7475	0.07878	0.5930 to 0.9020	0.007431		0.7571	0.07665	0.6069 to 0.9074	0.004869		0.5286	0.09192	0.3484 to 0.7088	0.7543
14,15-EET	0.6475	0.08991	0.4712 to 0.8238	0.1106		0.6976	0.087	0.5271 to 0.8682	**0.03045**	> 97.00	0.5167	0.09222	0.3359 to 0.6975	0.8551

9(10)-EpOME	0.74	0.07894	0.5853 to 0.8947	0.009437		0.7333	0.0801	0.5763 to 0.8904	**0.01062**	> 13906	0.5667	0.09128	0.3877 to 0.7456	0.4652
11,12-EET	0.5425	0.0929	0.3604 to 0.7246	0.6456		0.5905	0.09276	0.4086 to 0.7723	0.3217		0.569	0.09256	0.3876 to 0.7505	0.4495
8,9-EET	0.6	0.09092	0.4217 to 0.7783	0.2793		0.644	0.08924	0.4691 to 0.8190	0.1146		0.556	0.09146	0.3767 to 0.7352	0.54
5,6-EET	0.64	0.08851	0.4665 to 0.8135	0.1299		0.6821	0.08797	0.5097 to 0.8546	0.04607		0.5571	0.09335	0.3741 to 0.7402	0.5314
