# Supplementary material for: The oxylipin and endocannabidome responses in acute phase Plasmodium falciparum malaria in children
Source: Malar J. 2017 Sep 8;16:358. doi: 10.1186/s12936-017-2001-y (PMC5591560; doi:10.1186/s12936-017-2001-y)
Supplement: Supplementary file 15 — Additional file 15. Endocannabinoids levels (pM) in the blood serum samples from controls, uncomplicated malaria and severe malaria patients. [file 12936_2017_2001_MOESM15_ESM.pdf]

Additional file 15

The oxylipin and endocannabidome responses in acute phase *Plasmodium falciparum* malaria in children

Table. Endocannabinoids levels (pM) in the blood serum samples from controls, uncomplicated malaria and severe malaria patients. Shown are medians interquartile range (IQR) and total ranges for the lipids.

	Controls (n=20)			Uncomplicated malaria (n=20)			Severe malaria (n=21)		
**Compound**	**Median**	**IQR**	**Range**	**Median**	**IQR**	**Range**	**Median**	**IQR**	**Range**
**2AG**	13	39	6-51	48	48	9-82	49	84	16-235
**AEA**	1.7	2.4	0.1-4.8	1.6	4.5	0.5-13.4	2.9	11.6	0.3-31.4
**OEA**	2.3	1.6	1.1-4.4	7.6	5.0	3.5-18.0	10.1	8.9	4.1-18.9
**PEA**	3.4	4.8	1.0-8.1	9.2	8.4	5.2-19.7	13.0	10.6	7.8-21.8
**DEA**	0.2	0.2	0.1-0.6	0.5	0.4	0.2-1.0	0.7	0.5	0.3-1.1
**NAGLy**	11.0	6.8	2.9-29.8	6.2	4.9	2.1-20.2	5.1	9.3	2.3-58.3
**EPEA**	0.05	0.04	0.02-0.09	0.12	0.07	0.01-0.26	0.14	0.14	0.07-0.57
**DHEA**	0.3	0.5	0.2-1.2	0.8	1.8	0.3-3.0	1.4	1.7	0.3-3.5
**POEA**	0	0.3	0-0.4	0.2	0.5	0.1-1.6	0.4	0.6	0.0-2.1
**LEA**	1.7	2.7	0.2-4.6	2.1	2.8	0.7-8.1	2.3	2.3	0.9-5.1
**$PGF_{2\alpha}$-EA**	0.006	0.003	0.003-0.009	0.005	0.007	0-0.009	0.005	0.004	0.0-0.14
**PGE_2-EA**	0.004	0.006	0.0-0.028	0.006	0.007	0-0.029	0.006	0.031	0.0-0.049
