# Supplementary material for: The oxylipin and endocannabidome responses in acute phase Plasmodium falciparum malaria in children
Source: Malar J. 2017 Sep 8;16:358. doi: 10.1186/s12936-017-2001-y (PMC5591560; doi:10.1186/s12936-017-2001-y)
Supplement: Supplementary file 16 — Additional file 16. OPLS-DA correlation loadings from the uncomplicated versus controls and severe versus controls models. [file 12936_2017_2001_MOESM16_ESM.pdf]

Additional file 16

The oxylipin and endocannabidome responses in acute phase *Plasmodium falciparum* malaria in children

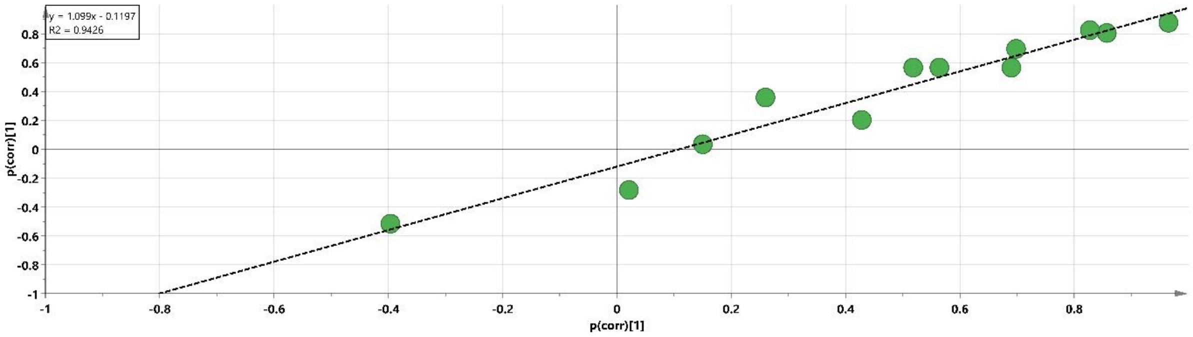

Figure. OPLS-DA correlation loadings (p(corr)) from the uncomplicated versus controls (X axis) and severe versus controls (Y axis) models; with dots symbolizing endocannabinoid species.
