# Supplementary material for: The oxylipin and endocannabidome responses in acute phase Plasmodium falciparum malaria in children
Source: Malar J. 2017 Sep 8;16:358. doi: 10.1186/s12936-017-2001-y (PMC5591560; doi:10.1186/s12936-017-2001-y)
Supplement: Supplementary file 17 — Additional file 17. Scatter plots for endocannabinoids that showed significant p-values in between group comparisons. [file 12936_2017_2001_MOESM17_ESM.pdf]

Additional file 17

The oxylipin and endocannabidome responses in acute phase *Plasmodium falciparum* malaria in children

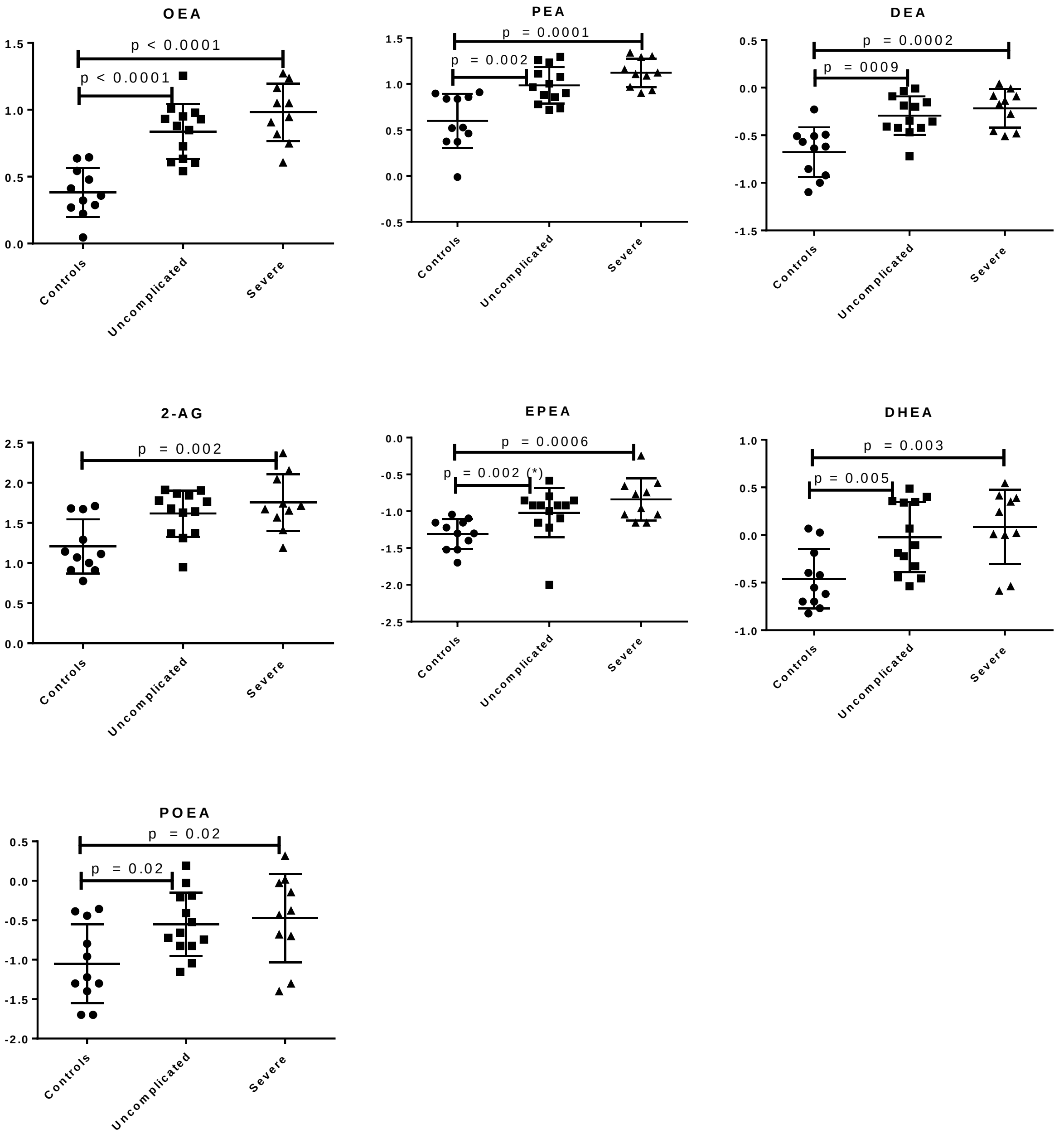

Figure. Scatter plots for endocannabinoids that showed significant p-values (t-test analysis with Welch’s correction) in between group comparisons (based on the log transformed data); (*) – Mann-Whitney test.
