# Supplementary material for: The oxylipin and endocannabidome responses in acute phase Plasmodium falciparum malaria in children
Source: Malar J. 2017 Sep 8;16:358. doi: 10.1186/s12936-017-2001-y (PMC5591560; doi:10.1186/s12936-017-2001-y)
Supplement: Supplementary file 18 — Additional file 18. Correlation of OEA and EPEA with parasitaemia values. [file 12936_2017_2001_MOESM18_ESM.pdf]

Additional file 18

The oxylipin and endocannabidome responses in acute phase *Plasmodium falciparum* malaria in children

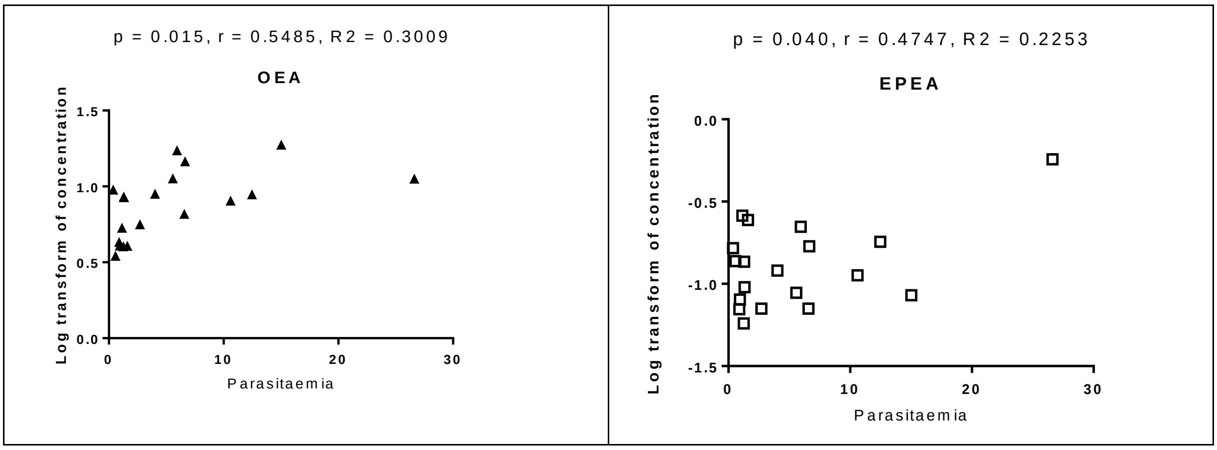

Figure. Correlation of OEA (left) and EPEA (right) with parasitaemia values.
