# Supplementary material for: The oxylipin and endocannabidome responses in acute phase Plasmodium falciparum malaria in children
Source: Malar J. 2017 Sep 8;16:358. doi: 10.1186/s12936-017-2001-y (PMC5591560; doi:10.1186/s12936-017-2001-y)
Supplement: Supplementary file 19 — Additional file 19. ROC curves results for endocannabinoids. [file 12936_2017_2001_MOESM19_ESM.pdf]

Additional file 19

The oxylipin and endocannabidome responses in acute phase *Plasmodium falciparum* malaria in children

Table. ROC curves results for endocannabinoids: area under the curve (AUC), standard error (std error), p value and Youden score (pM), in bold significant metabolites.

	**Controls vs Uncomplicated**					**Controls vs severe**					**Uncomplicated vs Severe**			
**Compound**	**AUC**	**Std error**	**95% CI**	**P value**	**Youden Score (nM)**	**AUC**	**Std error**	**95% CI**	**P value**	**Youden Score**	**AUC**	**Std error**	**95% CI**	**P value**
**2AG**	**0.95**	**0.050**	**0.855 - 1.05**	**0.001**	**> 19.98**	**0.988**	**0.020**	**0.948 -1.027**	**0.001**	> 21.42	0.5315	0.1241	0.2881 to 0.7748	0.7943
**AEA**	0.55	0.132	0.290 to 0.809	0.694		0.646	0.123	0.405 to 0.886	0.260		0.6231	0.1264	0.3752 to 0.8709	0.3211
**OEA**	**0.94**	**0.047**	**0.845 to 1.03**	**0.0003**	**> 4.870**	**0.893**	**0.088**	**0.721 to 1.07**	**0.002**	**> 5.015**	0.6846	0.1157	0.4578 to 0.9114	0.1367
**PEA**	**0.86**	**0.075**	**0.709 to 1.00**	**0.003**	**> 7.900**	**0.901**	**0.087**	**0.730 to 1.07**	**0.001**	**> 7.920**	0.7308	0.1051	0.5248 to 0.9367	0.06287
**DEA**	**0.909**	**0.065**	**0.782 to 1.04**	**0.001**	**> 0.3300**	**0.860**	**0.092**	**0.678 to 1.04**	**0.004**		0.5923	0.1281	0.3411 to 0.8435	0.4568
**NAGLy**	0.657	0.117	0.428 to 0.886	0.192		0.711	0.119	0.477 to 0.944	0.094		0.6014	0.125	0.3563 to 0.8465	0.4009
**EPEA**	**0.860**	**0.083**	**0.698 to 1.02**	**0.003**	**> 0.0950**	**0.855**	**0.090**	**0.679 to 1.03**	**0.005**	**> 0.0850**	0.5594	0.1269	0.3107 to 0.8082	0.6224
**DHEA**	**0.90**	**0.065**	**0.774 to 1.03**	**0.002**	**> 0.4350**	**0.838**	**0.098**	**0.647 to 1.03**	**0.011**	**> 0.8250**	0.528	0.1263	0.2804 to 0.7755	0.8167
**POEA**	**0.76**	**0.107**	**0.549 to 0.966**	**0.036**	**> 0.1700**	0.723	0.112	0.505 to 0.942	0.076	**> 0.1800**	0.535	0.1273	0.2854 to 0.7845	0.7721
**LEA**	0.62	0.117	0.392 to 0.853	0.311		0.537	0.127	0.289 to 0.786	0.768		0.5804	0.1213	0.3427 to 0.8181	0.5053

**$PGF_{2\alpha}$-EA**	0.62	0.117	0.389 to 0.848	0.325		0.632	0.127	0.384 to 0.880	0.294		0.514	0.1237	0.2715 to 0.7565	0.9078
**PGE_2-EA**	0.52	0.125	0.279 to 0.768	0.852		0.509	0.138	0.239 to 0.779	0.944		0.5105	0.1328	0.2502 to 0.7708	0.9308
